# Phase Variation in HMW1A Controls a Phenotypic Switch in Haemophilus influenzae Associated with Pathoadaptation during Persistent Infection

**DOI:** 10.1128/mBio.00789-21

**Published:** 2021-06-22

**Authors:** Ariadna Fernández-Calvet, Begoña Euba, Celia Gil-Campillo, Arancha Catalan-Moreno, Javier Moleres, Sara Martí, Alexandra Merlos, Jeroen D. Langereis, Francisco García-del Portillo, Lauren O. Bakaletz, Garth D. Ehrlich, Eric A. Porsch, Margarita Menéndez, Joshua C. Mell, Alejandro Toledo-Arana, Junkal Garmendia

**Affiliations:** a Instituto de Agrobiotecnología, Consejo Superior de Investigaciones Científicas (IdAB-CSIC)-Gobierno de Navarra, Mutilva, Spain; b Centro de Investigación Biomédica en Red de Enfermedades Respiratorias (CIBERES), Madrid, Spain; c Microbiology Department, Hospital Universitari Bellvitge, University of Barcelona, IDIBELL, Barcelona, Spain; d Department of Pathology and Experimental Therapeutics, IDIBELL-University of Barcelona, L’Hospitalet de Llobregat, Barcelona, Spain; e Section Pediatric Infectious Diseases, Laboratory of Medical Immunology, Radboud Institute for Molecular Life Sciences, Radboudumc, Nijmegen, The Netherlands; f Section Pediatric Infectious Diseases, Laboratory of Medical Immunology, Radboud Center for Infectious Diseases, Radboudumc, Nijmegen, The Netherlands; g Laboratorio de Patógenos Bacterianos Intracelulares, Centro Nacional de Biotecnología (CNB)-CSIC, Madrid, Spain; h Center for Microbial Pathogenesis, Abigail Wexner Research Institute at Nationwide Children’s Hospital, Columbus, Ohio, USA; i Department of Pediatrics, The Ohio State University College of Medicine, Columbus, Ohio, USA; j Department of Microbiology and Immunology, Drexel University College of Medicine, Philadelphia, Pennsylvania, USA; k Center for Genomic Sciences, Drexel University College of Medicine, Philadelphia, Pennsylvania, USA; l Center for Advanced Microbial Processing, Drexel University College of Medicine, Philadelphia, Pennsylvania, USA; m Center for Surgical Infections and Biofilms, Institute for Molecular Medicine and Infectious Disease, Drexel University College of Medicine, Philadelphia, Pennsylvania, USA; n Department of Otolaryngology, Head and Neck Surgery, Drexel University College of Medicine, Philadelphia, Pennsylvania, USA; o Department of Pediatrics, The Children’s Hospital of Philadelphia, Philadelphia, Pennsylvania, USA; p Instituto de Química Física Rocasolano (IQFR-CSIC), Madrid, Spain; Yale School of Public Health

**Keywords:** *Haemophilus influenzae*, allelic variant, phase variation, epithelial hyperinvasion, biofilm, persistence, lifestyle switch, pathoadaptation, high-molecular-weight (HMW), chronic obstructive pulmonary disease (COPD)

## Abstract

Genetic variants arising from within-patient evolution shed light on bacterial adaptation during chronic infection. Contingency loci generate high levels of genetic variation in bacterial genomes, enabling adaptation to the stringent selective pressures exerted by the host. A significant gap in our understanding of phase-variable contingency loci is the extent of their contribution to natural infections. The human-adapted pathogen nontypeable Haemophilus influenzae (NTHi) causes persistent infections, which contribute to underlying disease progression. The phase-variable high-molecular-weight (HMW) adhesins located on the NTHi surface mediate adherence to respiratory epithelial cells and, depending on the allelic variant, can also confer high epithelial invasiveness or hyperinvasion. In this study, we characterize the dynamics of HMW-mediated hyperinvasion in living cells and identify a specific HMW binding domain shared by hyperinvasive NTHi isolates of distinct pathological origins. Moreover, we observed that HMW expression decreased over time by using a longitudinal set of persistent NTHi strains collected from chronic obstructive pulmonary disease (COPD) patients, resulting from increased numbers of simple-sequence repeats (SSRs) downstream of the functional P2*_hmw1A_* promoter, which is the one primarily driving HMW expression. Notably, the increased SSR numbers at the *hmw1* promoter region also control a phenotypic switch toward lower bacterial intracellular invasion and higher biofilm formation, likely conferring adaptive advantages during chronic airway infection by NTHi. Overall, we reveal novel molecular mechanisms of NTHi pathoadaptation based on within-patient lifestyle switching controlled by phase variation.

## INTRODUCTION

Human-adapted pathogens take up long-term residence in or on the human body as part of the normal human microbiome but can also cause acute and chronic infections depending on conditions. This is the case for chronic obstructive pulmonary disease (COPD), a major leading cause of death globally, whose primary risk factor is smoking, characterized by irreversible airflow obstruction presenting emphysema, fibrosis, neutrophil airway infiltration, mucus hypersecretion, inflammation, and long-term lower airway colonization by pathogens (1–3). Observing the within-host evolution of pathogens over the course of long-term infection allows us to witness the emergence of adaptations that enable pathogens to persist, which could indicate the molecular mechanisms that drive chronic disease (4–7).

Nontypeable Haemophilus influenzae (NTHi) is normally an asymptomatic colonist of the human upper airways, especially in children, but also a leading cause of chronic opportunistic infections in the lower airways of COPD patients (8–10). As a facultative intracellular pathogen, NTHi invades a variety of human cell types (11–19), and epithelial cell invasion may allow NTHi to temporarily evade the immune system (20). After invasion, bacterial cells may remain nonproliferative and reside within membrane-bound vacuoles with features of late endosomes (21, 22), be free within the cytoplasm (23), develop intracellular bacterial communities in the cytoplasm (14), or eventually die (24).

Like many other bacteria, NTHi strains show high genomic and phenotypic diversity, and individuals tend to be colonized by many strains over time (3, 8, 25). Whole-genome sequencing of longitudinally collected NTHi strains from COPD patients revealed rapid genetic changes in clonally related strains due to natural transformation events (bringing recombination tracts from relatives into the chromosome), point mutations and chromosomal rearrangements, and phase-variable changes in simple-sequence repeats (SSRs) at contingency loci, which produce reversible and high-frequency genetic changes in specific genomic loci (26). Recurrent genomic changes affecting specific NTHi genes indicate the underlying selection pressures that shape pathogen adaptation within the COPD lung environment (4, 27–29).

We recently found evidence that the high-molecular-weight (HMW) adhesin HMW1A is implicated in NTHi pathoadaptation, identifying recurrent genetic changes arising in COPD patients over time (4). In a previous study using natural transformation to map NTHi genes involved in intracellular invasion (30), we had found that specific alleles of the *hmw1A* gene conferred novel high airway epithelial cell invasiveness, i.e., a hyperinvasive phenotype: natural transformation of the whole *hmw1* locus from the clinical isolate 86-028NP into low-invasion laboratory isolate RdKW20 (or of the *hmw1A* allele into a moderately invasive clinical isolate, Hi375, substituting for its native *hmw2A* allele) resulted in recombinants with ∼500- to 1,000-fold increased invasion into airway epithelial cells.

Genes encoding the HMW adhesins and their accessory proteins are found in 40 to 75% of NTHi strains and are nearly always found as two paralogous loci located at different positions of the chromosome, *hmw1ABC* and *hmw2ABC* (31–34). The *hmwA* genes display extensive diversity within and among strains, and their expression is phase variable due to tandem heptanucleotide SSRs [(5′-ATCTTTC)*_n_*] located between the P2 and P1 promoter regions (31, 32, 35–38). As SSR numbers increase, *hmwA* transcript and HMW protein levels decrease, with unclear effects on the NTHi-host interplay (36, 38, 39).

In this study, we tackled open questions regarding HMW-mediated NTHi hyperinvasion of airway epithelial cells, including examining *in vivo* bacterial entry dynamics and intracellular fate after hyperinvasion. Moreover, we hypothesized that HMW variants conferring hyperinvasion might contribute to successful chronic infection or be modulated by phase variation over time. Therefore, we examined the association of HMW-mediated hyperinvasion with specific HMW allelic variants in the previously sequenced longitudinal set of COPD strains (4), including its regulation by phase variation in the *hmw1* promoter. Together, the data from this work revealed a within-patient switch during persistent infection, from high cell invasiveness to biofilm growth, controlled by phase variation-reduced expression of the HMW1 adhesin.

## RESULTS

### Dynamics of NTHi epithelial hyperinvasion mediated by HMW1A_86-028NP_ and intracellular fate of bacterial aggregates.

The HMW1A_86-028NP_ adhesin allele was previously isolated as a key factor for epithelial hyperinvasion by the H. influenzae 86-028NP strain (originally isolated from a child with severe otitis media [OM] [40]) using natural transformation. Fluorescence microscopy showed Lamp-1 vesicle reorganization around large groups of internalized hyperinvasive *hmw1A* (*hmw1A*_hyper_) bacteria after cell invasion, contrasting with the vesicles containing singlet bacteria seen in lowly or moderately invasive strains (30). However, the *in vivo* dynamics of bacterial entry had not been observed. To monitor H. influenzae entry within airway epithelial cells, we performed time-lapse fluorescence microscopy during intracellular invasion into A549 cells after first loading acidic compartments with Lysotracker red DN99 (21). Next, cells were infected with a green fluorescent protein (GFP)-expressing 86-028NP strain, and imaging was initiated at 15 min postinfection (set at 0 min for simplicity).

**FIG 1:**
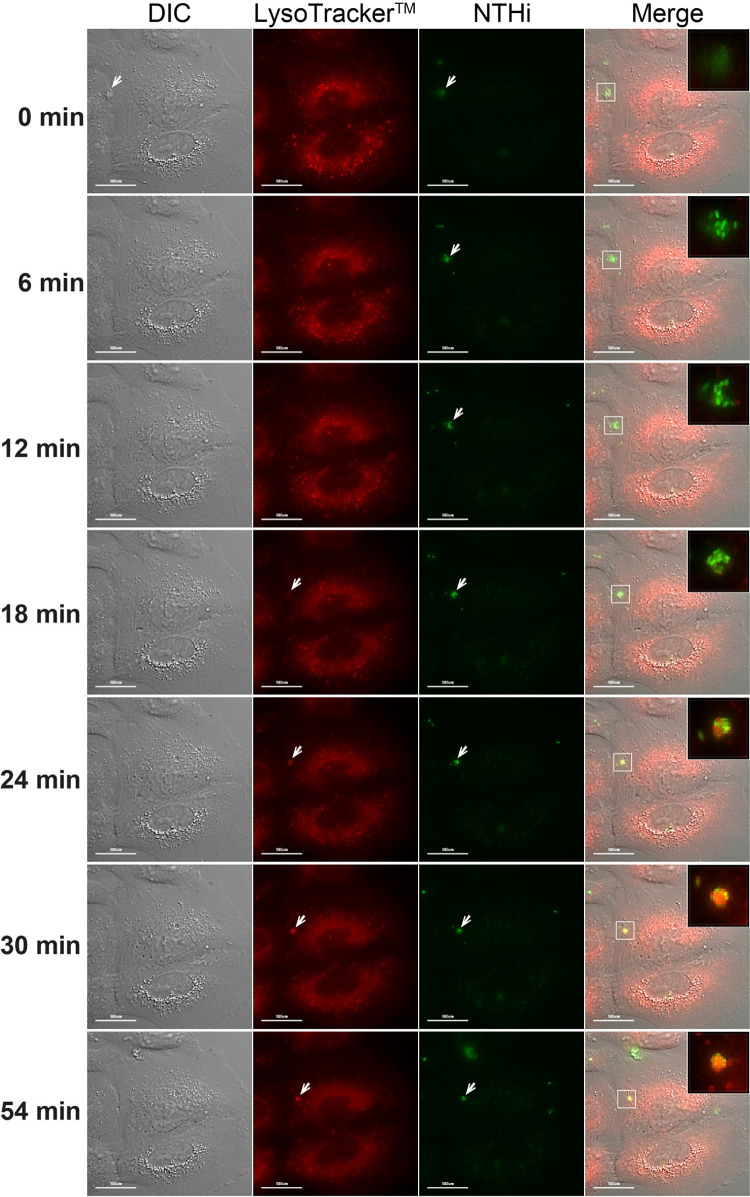
Time-lapse fluorescence microscopy of living A549 cells infected by NTHi. Stills from time-lapse imaging of A549 cells loaded with Lysotracker (red) and infected with NTHi GFP-86-028NP (green) are shown. The imaging starting point, referred to as 0 min, corresponding to bacteria adhered to the cell surface, was initiated at 15 min postinfection. Cells were imaged every 3 min until 54 min. Differential interference contrast (DIC) images are shown in the left column, where a group of bacteria is visualized extracellularly at 0 min (white arrows), while at 6 min, it has been internalized. Bacteria are seen to colocalize with Lysotracker from 18 min (white arrows). The elapsed time is shown on the left of each image. The merged panels at the right show a zoomed detail of the bacterial group (for a video, see Movie S1 in the supplemental material).

**FIG 2:**
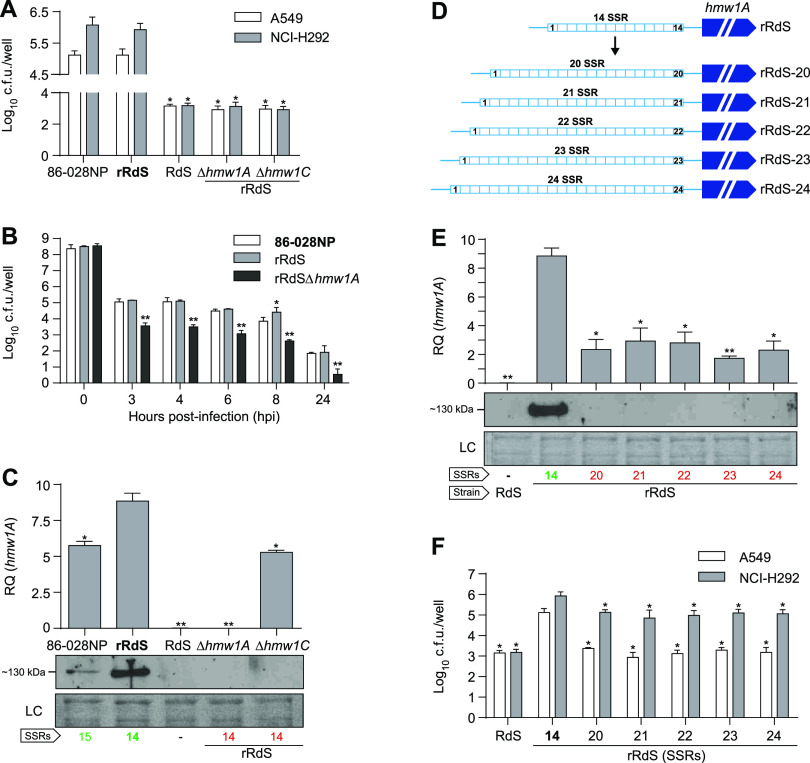
NTHi 86-028NP *hmw1A* and *hmw1C* genes contribute to the HMW hyperinvasive phenotype. Strains used as references for statistical comparisons are indicated in boldface type in each panel. (A) Inactivation of *hmw1A* and *hmw1C* genes reduced NTHi rRdS entry into A549 and NCI-H292 cells (*, *P* < 0.0001). Results of at least three independent experiments (*n* ≥ 3) in triplicate are shown as the mean log_10_ CFU per well ± standard deviations (SD). Statistical comparisons of the means were performed by one-way analysis of variance (ANOVA) and Dunnett’s multiple-comparison test. (B) Quantification of intracellular NTHi 86-028NP, rRdS, and rRdS Δ*hmw1A* bacterial counts in A549 cells over time. Strain comparisons showed a significant reduction in the RdS Δ*hmw1A* invasion rate at 3, 4, 6, 8, and 24 hpi (**, *P* < 0.0001). In all cases, a reduction of intracellular bacterial counts was detected at the assay endpoint compared to their respective initial titers. Results of three independent experiments (*n* = 3) in triplicate are shown as the mean log_10_ CFU per well ± SD. Statistical comparisons of means were performed by two-way ANOVA and Sidak’s multiple-comparison test. (C) Effect of *hmw1A* and *hmw1C* gene inactivation on the expression of the *hmw1A* gene. The expression of the *hmw1A* gene was undetectable in the rRdS Δ*hmw1A* and RdS strains (**, *P* < 0.0001) and lower in rRdS Δ*hmw1C* (*, *P* < 0.005) than in the rRdS WT strain. The expression level of the *hmw1A* gene was lower in 86-028NP than in the rRdS strain (*, *P* < 0.005). Results of at least two independent experiments (*n* ≥ 2) in triplicate are shown as the mean RQ (relative quantification, 2^−Δ^*^CT^* × 10) values ± standard errors of the means (SEM). Statistical comparisons of means were performed by one-way ANOVA and Dunnett’s multiple-comparison test. The bottom panel shows detection of HMW1_86-028NP_ by Western blotting; the SSR number at the *hmw1A* promoter regions is indicated (green indicates that HMW1A is immunodetected, and red indicates that HMW1A is not immunodetected). (D) Diagram illustrating the generated rRdS derivative strains, where the *hmw1A* promoter region presents a range of SSRs from 20 to 24. (E and F) Assays performed with rRdS derivative strains containing a variable number of SSRs in the *hmw1A* promoter region. RdS was used as a negative control. (E) Increased number of 7-bp tandem repeats reduced *hmw1A* gene expression (*, *P* < 0.005; **, *P* < 0.001). Results of at least two independent experiments (*n* ≥ 2) in triplicate are shown as the mean RQ (2^−Δ^*^CT^* × 10) values ± SEM. The bottom panel shows that an increased number of SSRs reduced HMW1A protein to a nondetectable level. The number of SSRs in the *hmw1* promoter is indicated in green (positive protein detection) or red (negative protein detection). (F) An increased number of SSRs eliminates the rRdS epithelial hyperinvasion phenotype (*, *P* < 0.0001). Results of at least three independent experiments (*n* ≥ 3) in triplicate are shown as the mean log_10_ CFU per well ± SD. In panels E and F, statistical comparisons of means were performed by one-way ANOVA and Dunnett’s multiple-comparison test. At the bottom of panels C and E, immunoblots were performed by using primary guinea pig anti-HMW (gp85 antibody) and secondary goat anti-guinea pig-horseradish peroxidase (HRP) antibodies. Three independent experiments were performed (*n* = 3), and a representative image is shown. The corresponding Coomassie-stained gel portion is shown as a loading control (LC).

Image analysis revealed that once bacterial cells or cell aggregates adhered to the epithelial cell surface, they entered rapidly (between 0 and 6 min) (for representative images, see Fig. 1; for a video, see Movie S1 in the supplemental material). Internalization of both single bacterial cells and aggregated groups was observed, with the latter event being frequent. Once inside, colocalization with Lysotracker-loaded acidic compartments was seen from 18 min onwards. When observing internalized groups of bacteria, colocalization with Lysotracker and reorganization toward a compact bacterial aggregate increased over time. Intracellular bacterial CFU titers remained stable for more than 4 h postinfection (hpi) and then decreased over time, but viable intracellular bacteria remained present at 24 hpi (Fig. 2B, white bars, and Fig. S1). Consistent with previous observations (21), intracellular bacteria appeared to remain metabolically active inside acidic endosome-like compartments throughout the course of infection (Fig. S1).

10.1128/mBio.00789-21.2 FIG S1 NTHi intracellular fate in human airway epithelial cells. Representative fluorescence microscopy images of A549 cells infected with NTHi rRdS at the time points indicated are shown. NTHi remains metabolically active inside acidic endosomal compartments. (A) Location of NTHi inside acidic compartments. NTHi and host cell nuclei were stained with Hoechst 33342 (blue), acidic subcellular compartments were loaded with Lysotracker (red), and bacteria were labeled with rabbit anti-NTHi and donkey anti-rabbit-Cy2 (green) antibodies. (B) Location of fluorescence *in situ* hybridization (FISH)-positive NTHi inside Lamp-1-positive compartments. NTHi and host cell nuclei were stained with Hoechst 33342 (blue), subcellular compartments were labeled with mouse anti-Lamp-1 and donkey anti-mouse-Cy2 (green) antibodies, and metabolically active bacteria were labeled with the probes EUB338-Cy3 and GAM42a-Cy3 (red). Download FIG S1, TIF file, 2.3 MB. Copyright © 2021 Fernández-Calvet et al. 2021 Fernández-Calvet et al. https://creativecommons.org/licenses/by/4.0/ This content is distributed under the terms of the Creative Commons Attribution 4.0 International license.

10.1128/mBio.00789-21.10 MOVIE S1 Time-lapse fluorescence microscopy of NTHi GFP-86-028NP internalization in living A549 cells. Acidic subcellular compartments from A549 cells were loaded with Lysotracker (red) prior to NTHi 86-028NP(pRSM2211) (green) infection. Download Movie S1, MOV file, 2.4 MB. Copyright © 2021 Fernández-Calvet et al. 2021 Fernández-Calvet et al. https://creativecommons.org/licenses/by/4.0/ This content is distributed under the terms of the Creative Commons Attribution 4.0 International license.

Results from live imaging of the dynamics of NTHi invasion into airway epithelial cells support our previous speculation that HMW1_86-028NP_-mediated aggregates of bacterial cells invade as groups, different from invasion as single cells seen in strains with other alleles or no *hmw* loci (21, 30). Furthermore, a significant load of intracellular metabolically active bacterial cells continues to reside in acidified compartments with late endosome features after 24 hpi. This differs from previous results with moderately invasive otitis media isolate Hi375, in which intracellular bacteria after invasion typically occupy vesicles as single cells (21). This might indicate that intracellular bacterial cell aggregates survive longer than single-cell invaders, but direct comparison is complicated by the substantially fewer bacteria of Hi375 that are initially taken up by host cells.

### HMW1C and phase-variable expression of the *hmw1A* gene are required for epithelial hyperinvasion mediated by HMW1_86-028NP_.

The HMW1 adhesin is encoded by *hmw1A*, as part of the *hmw1ABC* locus (34). A prerequisite for HMW1 adhesin activity is its glycosylation at asparagine (Asn) residues by the glycosyltransferase HMW1C (41), and Asn glycosylation levels vary with changes in the ratio of HMW1A to HMW1C (42). However, genetic analysis is complicated because proteins encoded by the paralogous *hmw2BC* normally found in HMW-positive strains can substitute for those of *hmw1BC* (43). As a surrogate strain to dissect the hyperinvasive *hmw1A*_86-028NP_ allele, we used a previously generated recombinant strain, rRdS, in which *hmw1ABC*_86-028NP_ was inserted into the poorly invasive laboratory strain RdKW20 by natural transformation, thereby conferring an invasion and intracellular life phenotype comparable to that of the hyperinvasive 86-028NP strain (Fig. 2A and B) (30). Since RdKW20 naturally lacks both the *hmw1* and *hmw2* loci and is also highly naturally transformable (in contrast to 86-028NP [44]), rRdS allowed us to uncouple *hmw1* from *hmw2* and provide for easier genetic manipulations and simpler construct design (engineered strains shown in Table 1).

**TABLE 1:** Bacterial strains used in this study

Strain(s)	Description	Source or reference
E. coli TOP10	Cloning strain; F^−^ *mcrA* Δ(*mrr-hsdRMS*-*mcrBC*) φ80*lacZ*ΔM15 Δ*lacX74 recA1 araD139* Δ(*ara leu*)*7697 galU galK rpsL* (Str^r^) *endA1 nupG*	Thermo Fisher Scientific

H. influenzae		
86-028NP/P190	Clinical isolate, otitis media origin, hyperinvasive; *hmw1^+^ hmw2^+^*	40
86-028NP(pRSM2211)/P561	86-028NP derivative, transformed with pRSM2211, GFP-expressing strain	63
RdS/P532	Rd Spec^r^; made by transforming P189 with P192 DNA, selecting for Spec^r^, and screening against other MAP7 resistance alleles	30
rRdS/P540	Rd Spec^r^; recombinant clone Nal^r^ s2, genotype B (rRdS); this strain acquired the *hmw1ABC* locus and the corresponding promoter region from strain 86-028NP by natural transformation	30
rRdS Δ*hmw1A*::*ermC*/P1025	rRdS derivative where the *hmw1A* gene was inactivated by homologous recombination with a Δ*hmw1A*::*ermC* cassette; Erm^r^	This study
rRdS Δ*hmw1C*::*ermC*/P1016	rRdS derivative where the *hmw1C* gene was inactivated by homologous recombination with a Δ*hmw1C*::*ermC* cassette; Erm^r^	This study
rRdS-20/P1068	rRdS derivative where the *hmw1A* gene promoter region has 20 heptanucleotide tandem repeats; Erm^r^	This study
rRdS-21/P1072	rRdS derivative where the *hmw1A* gene promoter region has 21 heptanucleotide tandem repeats; Erm^r^	This study
rRdS-22/P1070	rRdS derivative where the *hmw1A* gene promoter region has 22 heptanucleotide tandem repeats; Erm^r^	This study
rRdS-23/P1067	rRdS derivative where the *hmw1A* gene promoter region has 23 heptanucleotide tandem repeats; Erm^r^	This study
rRdS-24/P1066	rRdS derivative where the *hmw1A* gene promoter region has 24 heptanucleotide tandem repeats; Erm^r^	This study
P667, P668, P669	NTHi isolates from COPD patient 13, belonging to CT 3	4
P641, P642	NTHi isolates from COPD patient 10, belonging to CT 18	4
P617	NTHi isolate from COPD patient 6, belonging to CT 44	4
P634, P635, P636, P637	NTHi isolates from COPD patient 9, belonging to CT 44	4
P651, P652, P653, P654	NTHi isolates from COPD patient 10, belonging to CT 73	4
PittEE/P1078	Clinical isolate, otitis media origin; *hmw1^+^ hmw2^+^*	49
RdKW20[pTBH03-P2-(SSR)_13_-P1]/P1118	RdKW20 derivative, transformed with a GFP transcriptional reporter plasmid carrying the P*_hmw1-86-028NP_* promoter region with 13 heptanucleotide tandem repeats [(SSR)_13_] between the predicted P2 and P1 regions; Erm^r^	This study
RdKW20[pTBH03-P2-(SSR)_23_-P1]/P1119	RdKW20 derivative, transformed with a GFP transcriptional reporter plasmid carrying the P*_hmw1-86-028NP_* promoter region with 23 heptanucleotide tandem repeats [(SSR)_23_] between the predicted P2 and P1 regions; Erm^r^	This study
RdKW20(pTBH03-P2)/P1120	RdKW20 derivative, transformed with a GFP transcriptional reporter plasmid carrying a region of the P*_hmw1-86-028NP_* promoter containing the predicted P2 region; Erm^r^	This study
RdKW20(pTBH03-P1)/P1121	RdKW20 derivative, transformed with a GFP transcriptional reporter plasmid carrying a region of the P*_hmw1-86-028NP_* promoter containing the predicted P1 region; Erm^r^	This study
RdKW20[pTBH03-P2-(SSR)_14_]/P1114	RdKW20 derivative, transformed with a GFP transcriptional reporter plasmid carrying a region of the P*_hmw1-86-028NP_* promoter containing the predicted P2 region and 14 heptanucleotide tandem repeats [(SSR)_14_]; Erm^r^	This study
RdKW20[pTBH03-P2-(SSR)_24_]/P1115	RdKW20 derivative, transformed with a GFP transcriptional reporter plasmid carrying a region of the P*_hmw1-86-028NP_* promoter containing the predicted P2 region and 24 heptanucleotide tandem repeats [(SSR)_24_]; Erm^r^	This study
RdKW20[pTBH03-(SSR)_13_-P1]/P1116	RdKW20 derivative, transformed with a GFP transcriptional reporter plasmid carrying a region of the P*_hmw1-86-028NP_* promoter containing 13 heptanucleotide tandem repeats [(SSR)_13_] and the predicted P1 region; Erm^r^	This study
RdKW20[pTBH03-(SSR)_24_-P1]/P1117	RdKW20 derivative, transformed with a GFP transcriptional reporter plasmid carrying a region of the *P_hmw1-86-028NP_* promoter containing 24 heptanucleotide tandem repeats [(SSR)_24_] and the predicted P1 region; Erm^r^	This study

Confirming the role of HMW1 and the requirement for glycosylation by HMW1C for the expression of mature protein, we generated and tested rRdS Δ*hmw1A* and rRdS Δ*hmw1C* mutants. Both mutants lost the hyperinvasion phenotype in two cell types, behaving similarly to the negative control lacking *hmw* loci, RdS (Fig. 2A). Detection of HMW by immunoblotting showed that deletion of *hmw1C* led to undetectable HMW1A protein, although *hmw1A* transcript levels were only partially diminished (comparable to the transcript levels in 86-028NP, which expressed HMW1A protein and had invasion rates similar to those of rRdS) (Fig. 2C). These results confirm that HMW1A_86-028NP_ mediates hyperinvasion and that HMW1C-mediated glycosylation protects HMW1A against premature degradation (41).

**FIG 3:**
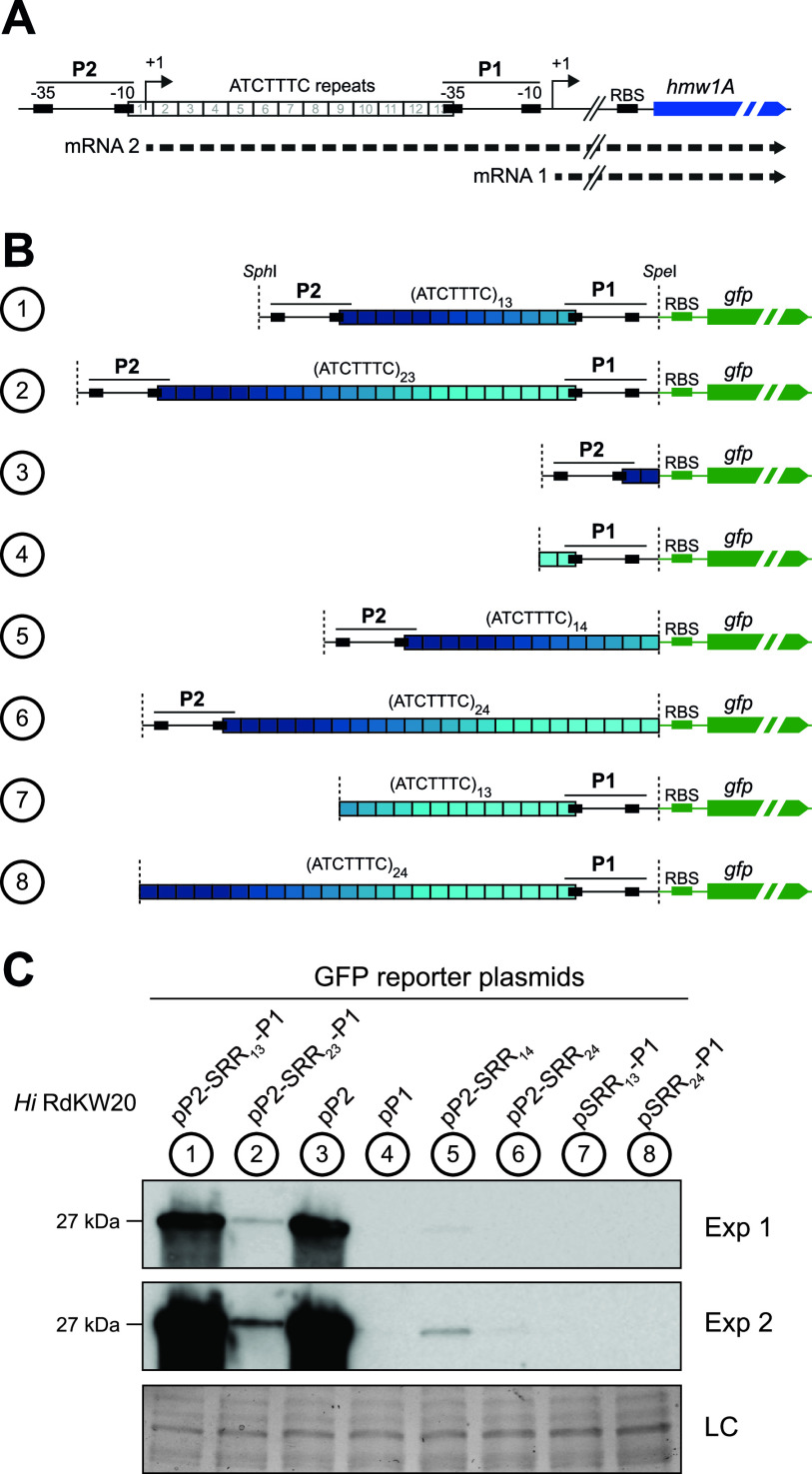
Variations in the number of SSRs affect P2*_hmw1A_* promoter activity. (A) Representation of the *hmw1A* promoter region with two possible transcriptional start sites, P2 and P1, separated by a tract of 5′-ATCTTTC repeats. The −35 box of the P1 promoter shares some nucleotides with the last repeat, while the −10 box of the P2 promoter includes some nucleotides from the first repeat (36). Transcription from these promoters could generate two alternative transcripts: P2 would generate a transcript including the SSRs in its 5′ UTR (mRNA 2), and P1 would produce a transcript lacking the SSRs (mRNA 1). RBS, ribosome binding site. (B) Diagram illustrating the panel of reporter plasmids including different *hmw1A* promoter regions fused to the *gfp* gene. (C) GFP was detected by using primary mouse anti-GFP and secondary goat anti-mouse IgG-HRP antibodies. Four independent experiments were performed (*n* = 4), and a representative image is shown. Exp 1 and Exp 2 indicate two different exposure times, 2 and 32 s, respectively. The corresponding Coomassie-stained gel portion is shown as a loading control (LC). *Hi*, H. influenzae.

Notably, HMW1A transcript and protein expression levels were significantly higher in the rRdS recombinant than in the 86-028NP parent strain (Fig. 2C). Although this expression difference might be a consequence of other differences in the genetic backgrounds or the absence of the paralogous *hmw2* loci, Sanger sequencing identified a difference in the counts of heptameric repeats in the SSRs upstream of *hmw1* in the two strains (between the P2 and P1 promoters) (Fig. 3A), with rRdS carrying 14 heptamers, compared to the parent 86-028NP strain’s 15 heptamers (and in contrast to the sequence reference for 86-028NP, which contains 17 heptamers [40]). This result is consistent with previous reports of phase-variable increases in copy numbers leading to decreased HMW1 adhesin expression (36, 38, 39).

To confirm that *hmw1A* expression, and also the hyperinvasion phenotype conferred by the HMW1A_86-028NP_ protein allele, decreases with increased upstream SSR lengths, we generated a panel of rRdS derivative clones with SSR counts from 20 to 24 (Fig. 2D). Clones with 20 to 24 repeats had no detectable HMW1A protein expression and had low invasion levels comparable to those of the RdS control lacking *hmw1*, in contrast to the high HMW1A expression levels seen in the hyperinvasive rRdS recombinant with 14 repeats in the heptameric repeat upstream of its *hmw1* locus (Fig. 2E and F).

### HMW1 expression is driven by the P2 promoter and sensitive to increases in its heptameric SSR.

Two distinct transcriptional start sites have previously been identified at *hmw1* by primer extension (promoters P2 and P1, separated by a heptameric SSR, 5′-ATCTTTC-3′, that overlaps the −10 box of P2 and the −35 box of P1 on each end) (Fig. 3A) (36). Transcripts from P2 contain the heptameric SSR in the 5′ untranslated region (UTR), while those from P1 do not; also, the SSR count could affect transcriptional activity from either promoter. To clarify the role of each promoter and the effect of changes in the SSR length, we generated a set of transcriptional fusion reporter plasmids coupling variants of the *hmw1* promoter region to GFP, which varied the heptameric SSR count and configuration of P2 and P1 (Fig. 3B). As controls, we used the full *hmw1A* promoter region including both P2 and P1, separated by either 13 or 23 repeats.

Reporter plasmids were introduced into the RdKW20 strain, and heterologous GFP protein levels were detected by immunoblotting (Fig. 3C). Expression levels from control plasmids were substantially higher with only 13 repeats than with 23 (plasmid 1 [pP2-SSR_13_-P1] and plasmid 2 [pP2-SSR_23_-P1]), consistent with *hmw1A* chromosomal expression in rRdS derivatives. Expression from mutant constructs with only the P1 or P2 elements indicates that most or all HMW1A protein expression is driven by transcripts initiated at P2 (plasmid 3 [pP2] and plasmid 4 [pP1]). GFP protein levels were strongly decreased from P2-only fusion plasmids, with 24 versus 14 repeats (plasmid 6 [pP2-SSR_24_] and plasmid 5 [pP2-SSR_14_]), underlining the effect of increased SSR copies on the repression of HMW1A expression. No GFP was detected in P1-only reporter plasmids with either 13 or 24 repeats (plasmid 7 [pSSR_13_-P1] and plasmid 8 [pSSR_24_-P1]). Further supporting P2 as the transcriptional start for expressed HMW1A, alignment of the *hmw1* upstream promoter region showed more variation in P1, mainly due to variation in AGGG repeats in the putative P1 −10 box (Fig. S2). Nevertheless, when analyzing the repeat number effect on GFP expression from P2 by including 14 or 24 repeats, we observed lower GFP levels than those in the control plasmids including the entire region (plasmids 5 and 6 versus plasmids 1 and 2, respectively), suggesting the potential presence of posttranscriptional regulatory elements downstream of the SSR in the long 5′ UTR generated from P2.

10.1128/mBio.00789-21.3 FIG S2 Multiple-sequence alignment of the *hmw1A* promoter region present in strains 86-028NP, R4846, PittEE, P669 (CT 3), P641 (CT 18), P636 (CT 44), and P652 (CT 73). Polymorphisms are indicated in gray, 7-bp tandem repeats (5′-ATCTTTC) are in yellow, and the open reading frame (ORF) start codon is in green. Putative −35 and −10 promoter positions are underlined, putative transcription initiation sites (P2 and P1) are indicated in red with an arrow, and the variable P1 region with AGGG repeats is indicated in blue. Download FIG S2, TIF file, 1.7 MB. Copyright © 2021 Fernández-Calvet et al. 2021 Fernández-Calvet et al. https://creativecommons.org/licenses/by/4.0/ This content is distributed under the terms of the Creative Commons Attribution 4.0 International license.

In summary, the expression of HMW1 is primarily driven by the P2 promoter and strongly affected by SSR length, although whether this effect is transcriptional or posttranscriptional remains unknown.

### Natural variation in HMW adhesins among longitudinally collected persistent NTHi isolates.

To test for genetic changes at *hmw1* during long-term infection and whether the hyperinvasive HMW1A allele can be found in NTHi persistent isolates, we examined the distribution and sequence divergence of the *hmw1* and *hmw2* loci across a well-characterized genome-sequenced set of 92 strains collected from COPD sputum samples over time, 72 of which were grouped into 20 “persistent” clonal types (CTs) that consisted of at least 2 nearly identical strains isolated at different times from the same subject. We previously used this collection to identify NTHi within-lung pathoadaptation traits, searching for genes recurrently affected by mutations in distinct CTs and in different patients (4). As references for the HMW adhesin loci, we used the prototype *hmw1A* and *hmw2A* gene sequences from NTHi strain R2846, where the HMW adhesins were originally identified and have been most extensively characterized (33). BLASTN analysis against the 92 genome assemblies (4) identified both *hmw* loci in 40 isolates (43.5%), comparable to previous clinical isolate surveys that found between 40 and 75% of strains with HMW adhesins (32, 45). To focus on HMW-positive strains from persistent CTs with multiple isolates, we further focused on a set of 14 isolates of 4 CTs for which there was a finished genome assembly available (see Table 2) (numbers of single nucleotide polymorphisms [SNPs] in noncoding regions were 0 for CT 3, 10 for CT 18, 298 for CT 44, and 1 for CT 73; numbers of SNPs in coding regions, including those of high, moderate, and low impacts, were 11 for CT 3, 103 for CT 18, 3,120 for CT 44, and 6 for CT 73).

**TABLE 2:** Summary of HMW-related features in the NTHi clinical isolates used in this study

Strain^*d*^	WGS platform	Isolation date (yr-mo-day)	Patient ID	HMW1					HMW2				
				Proximal gene (5′)	No. of SSRs	Preprotein mol wt (kDa)^*a*^	Mature protein mol wt (kDa)^*a*^	Western blot result (mol wt [kDa]/intensity)^*c*^	Proximal gene (5′)	No. of SSRs	Preprotein mol wt (kDa)^*a*^	Mature protein mol wt (kDa)^*a*^	Western blot result (mol wt [kDa]/intensity)^*c*^
CT 3													
P667	Illumina	2010-02-13	13	*yrbI*	12	166.6	120	∼140/+++	*radA*	16	152.3	105.8	∼120/+
P668	Illumina	2010-05-24	13	*yrbI*	21	166.6	120	ND	*radA*	15	152.3	105.8	∼120/++
**P669**	PacBio	2010-08-16	**13**	*yrbI*	22	166.6	120	ND	*radA*	14	152.3	105.8	∼120/+++

CT 18													
**P641**	PacBio	2009-12-28	**10**	*yrbI*	19	150.2	103.8	∼125/+	*radA*	16	157.5	111.2	∼135/++
P642	PacBio	2010-01-19	10	*yrbI*	18	150.2	103.8	∼125/+	*radA*	17	157.5	111.2	∼135/+

CT 44													
P634	Illumina	2011-02-22	9	*radA*	20	172.4	125.9	∼145/+	*yrbI*	18	156.2	109.7	∼130/+
P635	Illumina	2011-03-08	9	*radA*	10	172.4	125.9	∼145/+++	*yrbI*	19	156.2	109.7	ND
**P636**	PacBio	2012-06-05	**9**	*radA*	18	172.4	125.9	∼145/+	*yrbI*	22	156.2	109.7	ND
P637	Illumina	2012-06-30	9	*radA*	17	172.4	125.9	∼145/+	*yrbI*	22	156.2	109.7	ND
P617	PacBio	2013-11-13	6	*radA*	25	172.4	125.9	ND	*yrbI*	27	156.1	109.6	ND

CT 73													
P651	Illumina	2013-05-06	10	*radA*	11	162.8	116.4	∼135/+++	*yrbI*	16	167.9	121.4	∼140/++
**P652**	PacBio	2013-05-18	**10**	*radA*	13	162.8	116.4	∼135/+++	*yrbI*	16	167.9	121.4	∼140/++
P653	Illumina	2013-06-20	10	*radA*	18	162.8	116.4	∼135/+	*yrbI*	17	167.9	121.4	∼140/++
P654	Illumina	2013-07-05	10	*radA*	18	162.8	116.4	∼135/+	*yrbI*	17	167.9	121.4	∼140/++

86-028NP				*yrbI*	15	154.4	108.1	∼130/+	*radA*	23	160.6	114.2	ND
RdS													
rRdS				*yrbI*	14	154.4	108.1	∼130/+++					
PittEE				*yrbI*	14	162.8	116.4	∼135/+++	*radA*	38	164.4	117.9	ND
R2846				*yrbI*	16	159.9	113.4	125/++^*b*^	*radA*	17	154.3	107.8	120/+++^*b*^

a Protein theoretical molecular weight.

b Barenkamp and Leininger (64).

c Intensity of the Western blot band corresponding to HMW1A or HMW2A (+, low; ++, intermediate; +++, high). ND, not detected.

d Boldface type indicates a reference strain considered for intra-CT statistical analyses.

**FIG 4:**
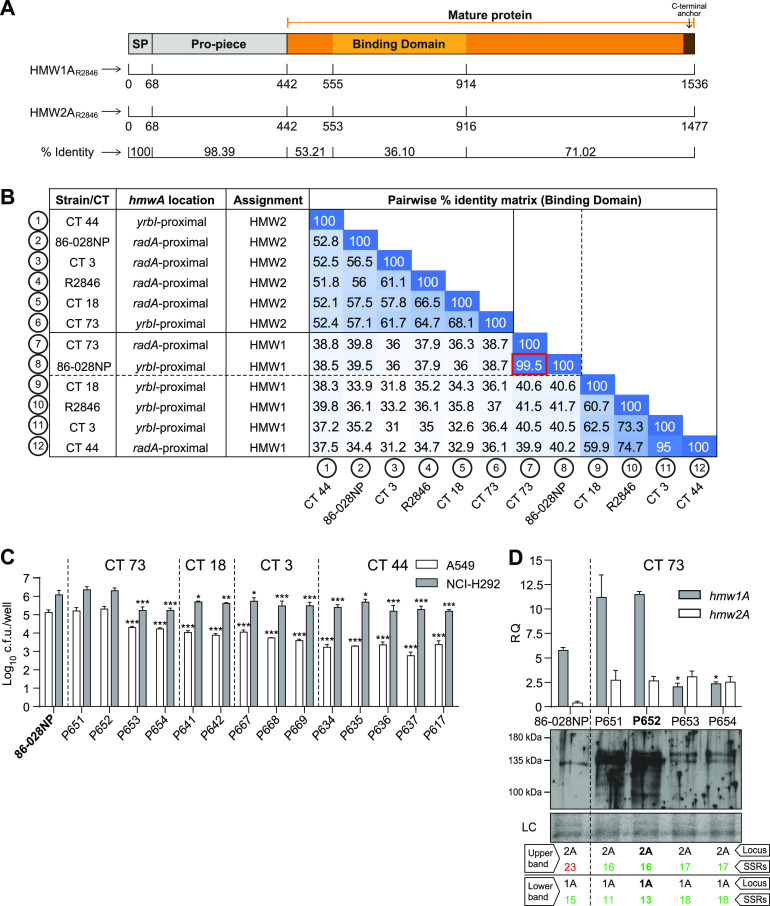
Distribution of the *hmw* loci and phase-variable hyperinvasion among serially collected NTHi clinical isolates. HMW1A and HMW2A binding domains from R2846 were used as reference sequences for HMW locus assignment by protein identity. (A) Schematic representation of the HMW1/HMW2 preproteins: signal peptide (residues 1 to 68), propiece (residues 69 to 441), and mature protein (residue 442 to the stop codon). The binding domain (residues 555 to 914 for HMW1A_R2846_ and residues 553 to 916 for HMW2A_R2846_) is the most variable sequence between them (36% identity). (B) Matrix of pairwise identities of the HMW binding domains of strains R2846 and 86-028NP and COPD clinical isolates of the indicated CTs. Locus 1/2 assignment was performed according to identity clustering (>50%). (C and D) Strains used as controls for statistical comparisons are indicated in boldface type in each panel. Strain 86-028NP was used as a hyperinvasion positive control. (C) A549 and NCI-H292 cell invasion by NTHi clinical isolates serially collected from COPD patients, belonging to CTs 73, 18, 3, and 44. Invasion levels were significantly lower (*, *P* < 0.05; **, *P* < 0.001; ***, *P* < 0.0001) than those shown by the 86-028NP strain, except for strains P651 and P652 (CT 73). Results of at least three independent experiments (*n* ≥ 3) in triplicate are shown as the mean log_10_ CFU per well ± SD. Statistical comparisons of means were performed by one-way ANOVA and Dunnett’s multiple-comparison test. (D) Phase variation in the *hmw1A* promoter region regulates the hyperinvasive phenotype. Assays were performed with CT 73 serial isolates where the SSR number in the *hmw1A* and *hmw2A* promoter regions has been determined. For intra-CT 73 comparisons, the strain labeled in boldface type was used as a reference. Significant intra-CT 73 differences in *hmw1A* gene expression were observed (*, *P* < 0.05). Statistical comparisons of means were performed by one-way ANOVA and Sidak’s multiple-comparison test. Results of at least two independent experiments (*n* ≥ 2) in triplicate are shown as the mean RQ (2^−Δ^*^CT^* × 10) values ± SEM. The bottom panel shows immunoblot detection of HMW1/2; the number of SSRs in the *hmw1* and *hmw2* promoter regions is indicated in green (positive protein detection) or red (negative protein detection). Immunoblots were performed by using primary guinea pig anti-HMW (gp85 antibody) and secondary goat anti-guinea pig-HRP antibodies. Two independent experiments were performed (*n* = 2); a representative image is shown. The corresponding Coomassie-stained gel portion is shown as a loading control (LC).

HMW1/2A consists of a signal peptide (SP), the propiece (PP) (containing the secretion domain that mediates interaction with the HMW1/2B outer membrane translocator, before cleavage and release at secretion), the mature HMW1/2A adhesin that contains the binding domain, and a small C-terminal anchor (46) (Fig. 4A). To distinguish between paralogous *hmw1* and *hmw2* loci in each of the 4 CTs, we compared their binding domains to those of R2846 (amino acids 555 to 914 in HMW1A and amino acids 553 to 916 in HMW2A [47]). To assign HMW adhesins as either HMW1 or HMW2, the percent identity was calculated between the 12 HMW binding domains of the six isolates with fully assembled genomes, and assignment was made, based on which had higher identity to which prototype adhesin in strain R2846. Assignment based on synteny is expected to be unreliable since although all *hmw* loci observed are adjacent to either the *yrbI* (NTHI1982) or *radA* (NTHI1453) gene, previous observations suggest that gene conversion can swap binding domains between loci (30). HMW binding domain sequences from hyperinvasive strain 86-028NP were included to test for similarity to protein alleles naturally occurring in COPD. Although HMW binding domains were highly diverse, as expected, most comparisons yielded a clear distinction between paralogs, with putative orthologs having >40 to 50% pairwise amino acid identity and putative paralogs having <40% pairwise identity, and their chromosomal locations were frequently shuffled (Fig. 4B). Clear exceptions are the putative HMW1 sequences from 86-028NP and four isolates of CT 73 with identical alleles (strains P651, P652, P653, and P654), which are 99.5% identical to each other but only slightly more similar to the prototype protein HMW1_R2846_ than to HMW2_R2846_ (41.5% versus 37.9%). These results suggest that the hyperinvasive HMW1 allele in strain 86-028NP (an otitis media isolate) can also be found in NTHi strains that have persisted in COPD infections.

When comparing the HMW1 variants present in 86-028NP and P651 to P654 (CT 73) to the rest of the tested strains, conservation was found across the signal peptide and propiece (Fig. S3, pink and green, respectively), with the adhesins themselves showing high divergence, particularly in the binding domain, as previously observed (47). Although the PP is well conserved, some differences were specific to HMW1A_86-028NP_ and HMW1A_CT73_. Since the crystal structure of the HMW1-PP from R2846 has been solved (46) (unlike the rest of the protein), we used structural homology modeling to visualize HMW1-PP differences. Figure S4 shows changes common and specific to the HMW1-PP from 86-028NP and CT 73: in the last turn of the β-helix (D424Y, F426S, K428G, D429N, N430D, I432A, and D434E) and in the length of the loop connecting the last two turns of the superhelix (G409del). When considering mature protein variants of HMW1 only, substantial divergence was observed due to SNPs and indels, rendering size variants ranging from 1,434 to 1,661 amino acids (Fig. S3). Furthermore, HMW1C Asn glycosylation across HMW1A_R2846_ adhesins has been experimentally demonstrated at NXS/T motifs (41, 42, 48), but the distinct HMW1A alleles show relatively poor conservation of these sites (70 to 91% across all variants [CT 3, 90.9%; CT 18, 75.8%; CT 44, 81.8%; CT 73 and 86-028NP, 69.7%]) (Fig. S3 [Asn positions that could be glycosylated are shown in red boldface type]).

10.1128/mBio.00789-21.4 FIG S3 Multiple-sequence alignment of HMW1 variants present in strains 86-028NP, R4846, and PittEE and strains of CT 3 (P669), CT 18 (P641), CT 44 (P636), and CT 73 (P652). Positions coincident with the consensus sequence of HMW1_R2846_ are indicated with a dot; nonconserved residues are indicated and shown in the corresponding position. Pink, signal peptide; green, propiece; white, mature protein; gray, binding domain within the mature protein. Polymorphisms shared by hyperinvasive strains are shown in blue. N-glycosylation sites (NXT/S) detected by mass spectrometry in HMW1_R2846_ (see references 15 to 17 in Text S1) are shown inside red boxes, with the Asn (N) residue labeled in red boldface type. Twenty-three N-glycosylation positions are predicted to be conserved in HMW1_R2846_, HMW1_86-028NP_, HMW1_PitEE_, and HMW1_CT73_ variants; HMW1_86-028NP_, HMW1_PitEE_, and HMW1_CT73_ variants are predicted to lose eight HMW1_R2846_ N-glycosylation sites. Download FIG S3, TIF file, 2.2 MB. Copyright © 2021 Fernández-Calvet et al. 2021 Fernández-Calvet et al. https://creativecommons.org/licenses/by/4.0/ This content is distributed under the terms of the Creative Commons Attribution 4.0 International license.

10.1128/mBio.00789-21.5 FIG S4 HMW-PP protein models for the P652 (CT 73), PittEE, and 86-028NP strains. (A) Cross section of the HMW-PP_R2846_ β-helix. (B) Longitudinal views of the HMW-PP_R2846_ β-helix (left) and protein surface (middle) and the overlap of both (right). Detail of the hydrophobic pocket is indicated with a black arrow. (C and D) Homology models for HMW-PP_CT73(P652)/PittEE_ (turquoise) (C) and HMW-PP_86-028NP_ (dark pink) (D) overlapping HMW-PP_R2846_ (gold). HMW-PP is 100% identical in strains P651 to P654 belonging to CT 3 and in the PittEE strain. For this reason, we refer to this homology model as HMW-PP_CT73/PittEE_. HMW-PP_CT73/PittEE_ and HMW-PP_86-028NP_ share C-terminal polymorphisms (indicated in black boldface type) at residues 424, 426, 428, 429, 430, 432, 434, and 409. Download FIG S4, TIF file, 2.5 MB. Copyright © 2021 Fernández-Calvet et al. 2021 Fernández-Calvet et al. https://creativecommons.org/licenses/by/4.0/ This content is distributed under the terms of the Creative Commons Attribution 4.0 International license.

In sum, HMW adhesins in longitudinally collected persistent NTHi isolates had high variability in chromosomal location and amino acid sequence, with evidence that strains of CT 73 could be hyperinvasive, like 86-028NP, due to their distinct *hmw1* allele.

### Serial CT 73 isolates from the same COPD patient lost expression of an epithelial hyperinvasion phenotype after SSR expansion downstream of the P2 promoter.

Since the four serial isolates of CT 73 (collected over ∼3 months from the same subject) had identical HMW1A protein sequences that were highly similar to that of hyperinvasion-inducing HMW1A_86-028NP_, we tested their epithelial invasion phenotypes in A549 and NCI-H292 cells by gentamicin protection assays, comparing them to 86-028NP and the 10 additional strains from HMW-positive multi-isolate CTs (Fig. 4C). Assays were performed using bacterial cultures normalized to the same CFU per milliliter, based on correlations with the optical density at 600 nm (OD_600_), thereby ensuring comparable multiplicities of infection (MOIs) among strains. Notably, CT 73 isolates from May 2013 (P651 and P652) had invasion rates comparable to those of hyperinvasive 86-028NP, whereas those collected later in June and July 2013 (see Table 2) had significantly lower invasion rates, comparable to those of the other HMW-positive strains from multi-isolate CTs collected from COPD patients.

All four CT 73 isolates had 100% identical HMW1 protein sequences, so we tested for correlations with the number of phase-variable heptameric SSRs downstream of the P2 site at the *hmw1* promoter, predicting increased copy numbers in the later-collected isolates (36, 38, 39). Sanger sequencing was used to genotype the SSRs at *hmw1* from all 14 strains in the four CTs since short-read sequencing had failed to confidently resolve differences in the long heptameric SSRs and often failed to distinguish reads from paralogous *hmw1* and *hmw2* loci. Strikingly, this revealed an expansion of the SSR from 11 to 18 heptamers at *hmw1* over time in the four CT 73 strains, which was associated with both a loss of hyperinvasion and decreased HMW1 mRNA and protein levels (Fig. 4C and D). SSR counts also varied within the other three CTs at both *hmw1* and *hmw2*, and increased SSR counts strongly correlated with decreased mRNA and protein levels at each adhesin paralog (Fig. S5A and B).

10.1128/mBio.00789-21.6 FIG S5 Phase variation in the *hmw1A* promoter region from CT 18, 3, and 44 COPD isolates. For intra-CT comparisons, strains labeled in boldface type were used as a reference. (A) Assays performed with the selected set of COPD serial isolates where the SSR number in the *hmw1A* and *hmw2A* promoters has been determined. Significant intra-CT differences in *hmw1A* gene expression were observed within CTs 3 and 44 (*, *P* < 0.0001). The results of at least two independent experiments (*n* ≥ 2) in triplicate are shown as the mean RQ (2^−Δ^*^CT^* × 10) values ± SEM. The bottom panel shows immunoblot detection of HMW1/2; the number of SSRs in the *hmw1* and *hmw2* promoter regions is indicated in green (positive protein detection) or red (negative protein detection). Statistical comparisons of means were performed by one-way ANOVA and Sidak’s multiple-comparison test. Immunoblots were performed by using primary guinea pig anti-HMW (gp85 antibody) and secondary goat anti-guinea pig-HRP antibodies. Three independent experiments were performed (*n* = 3), and a representative image is shown. (B) The corresponding Coomassie-stained gels are shown as a loading control (LC). (C) Biofilm formation by CT 18, 3, and 44 strains. Bacteria were cultured overnight at 37°C in microtiter plates under static conditions. The reduction of *hmwA* expression increased biofilm growth. Results of at least three independent experiments (*n* ≥ 3) in triplicate are shown as the mean OD_570_/OD_600_ ratio ± SEM. Statistical comparisons between means were performed by one-way ANOVA and Sidak’s multiple-comparison test (*, *P* < 0.05; **, *P* < 0.005; ***, *P* < 0.0001). Download FIG S5, TIF file, 1.5 MB. Copyright © 2021 Fernández-Calvet et al. 2021 Fernández-Calvet et al. https://creativecommons.org/licenses/by/4.0/ This content is distributed under the terms of the Creative Commons Attribution 4.0 International license.

**FIG 5:**
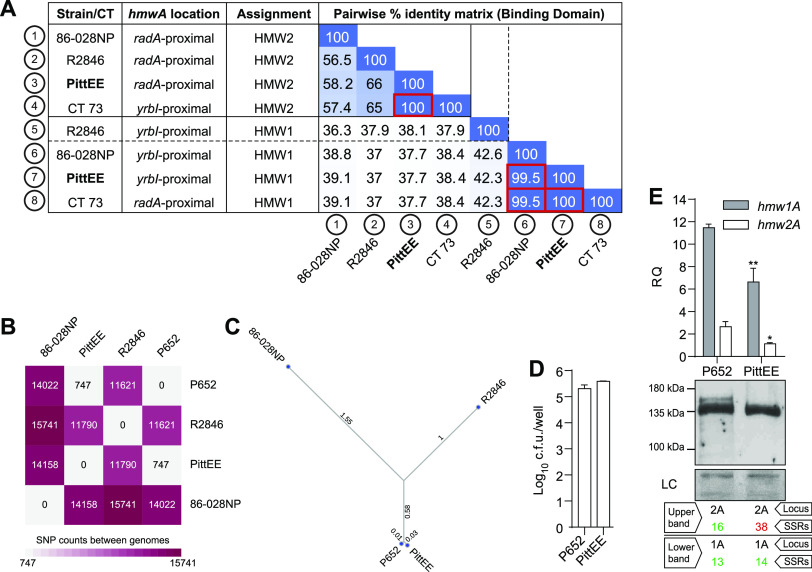
The NTHi PittEE strain has HMW-related hyperinvasive features. (A) Screening of HMW1_86-028NP_ binding domain homologs in publicly available NTHi genomes rendered >99% protein identity for HMW of the PittEE strain. A matrix of pairwise identities of the HMW binding domains of strain R2846, strain 86-028NP, CT 73, and strain PittEE is shown. Locus 1/2 assignment was performed according to identity clustering (>50%). (B and C) Whole-genome SNP-based phylogenetic analysis. (B) Matrix of SNP pair counts; (C) unrooted phylogenetic tree displaying branch lengths. (D) A549 cell invasion by P652 and PittEE. Results of at least three independent experiments (*n* ≥ 3) in triplicate are shown as the mean log_10_ CFU per well ± SD. Statistical comparisons of means were performed by a two-tailed *t* test. (E) Expression levels of the *hmw1A* and *hmw2A* genes were lower in PittEE than in the P652 strain (*, *P* < 0.05; **, *P* < 0.01). Statistical comparisons of means were done by a two-tailed *t* test. In all cases, results of at least two independent experiments (*n* ≥ 2) in triplicate are shown as the mean RQ (2^−Δ^*^CT^* × 10) values ± SEM. The bottom panel shows immunoblot detection of HMW1/2; the number of SSRs in the *hmw1* and *hmw2* promoter regions is indicated in green (positive protein detection) or red (negative protein detection). Two independent experiments were performed (*n* = 2); a representative image is shown. Immunoblots were performed by using primary guinea pig anti-HMW (gp85 antibody) and secondary goat anti-guinea pig-HRP antibodies. Three independent experiments were performed (*n* = 3); a representative image is shown. The corresponding Coomassie-stained gel portion is shown as a loading control (LC).

We aimed to confirm that *hmw1A* was responsible for the high invasiveness observed in CT 73 isolates P651 and P652, but natural transformation was negligible in these strains, preventing us from making the knockout. Thus, we sought evidence of an allele like *hmw1*_86-028NP_ in another strain and tested for hyperinvasiveness. We performed tBLASTn analysis using the HMW1_86-028NP_ binding domain as a query against all H. influenzae genomes available in the NCBI database, identifying the HMW1 binding domain of strain PittEE (HMW1_PittEE_) (49) as being 99.5% identical (Fig. 5A and Table S3). Strain PittEE (obtained from a child suffering from chronic otitis media with effusion) and the COPD isolate P652 were closely related (Fig. 5B and C). Further sequence analysis showed that HMW1_CT73_ and HMW1_PittEE_ are nearly identical, differing by only two polymorphisms outside the binding domain (N540S and K1475R). HMW1_86-028NP_, HMW1_CT73_, and HMW1_PittEE_ nicely clustered together, with up to 331 commonly shared and exclusive polymorphisms, 181 of them located in the binding domain compared to HMW1A_R2846_ (Fig. S3, shown in gray). Their predicted level of glycosylation conservation was only 70%, with eight Asn residues compatible with being glycosylated lost (Fig. S3). Although PittEE was also poorly transformable, epithelial invasion assays showed levels comparable to those of 86-028NP and P651/P652 (Fig. 4C and Fig. 5D). The *hmw1A*_PittEE_ and *hmw1A*_P652_ promoter regions contained 14 and 13 SSRs, respectively, and mRNA and protein expression levels showed lower *hmw1A* expression levels in PittEE (Fig. 5E).

10.1128/mBio.00789-21.8 TABLE S2 Primers used in this study. Download Table S2, DOCX file, 0.1 MB. Copyright © 2021 Fernández-Calvet et al. 2021 Fernández-Calvet et al. https://creativecommons.org/licenses/by/4.0/ This content is distributed under the terms of the Creative Commons Attribution 4.0 International license.

10.1128/mBio.00789-21.9 TABLE S3 tBLASTn analysis against published H. influenzae genomes using the HMW1_86-028NP_ binding domain (residues 542 to 908) as the query sequence. Download Table S3, DOCX file, 0.09 MB. Copyright © 2021 Fernández-Calvet et al. 2021 Fernández-Calvet et al. https://creativecommons.org/licenses/by/4.0/ This content is distributed under the terms of the Creative Commons Attribution 4.0 International license.

Together, these data support the association not only between a particular HMW1 variant and the hyperinvasive phenotype but also between the increased number of 7-bp repeats in the *hmw1A* promoter region and the decreased gene transcript and HMW protein levels, as observed in both the laboratory rRdS strain (Fig. 2) and the clinical CT 73 strain series (Fig. 4D).

### Phase-variable expression of HMW1_hyper_ mediates a switch between intracellular invasion and biofilm formation.

**FIG 6:**
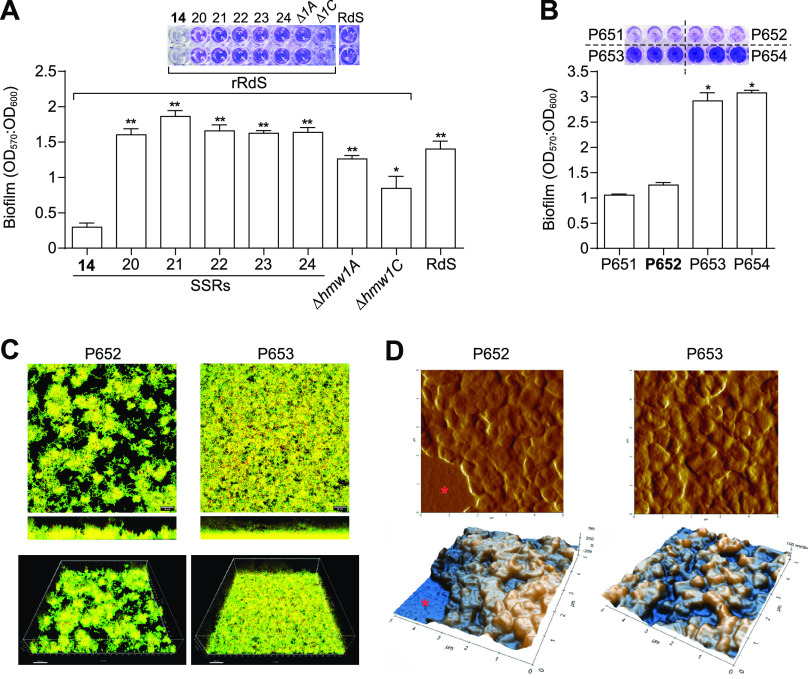
Phase variation in the *hmw1A* promoter region regulates NTHi biofilm growth. Strains used as controls for statistical comparisons are indicated in boldface type in each panel. (A) Biofilm formation of rRdS derivative strains, i.e., those containing a variable number of SSRs in the *hmw1A* promoter region (14 to 24 SSRs), and rRdS Δ*hmw1A* and rRdS Δ*hmw1C* mutants. RdS was used as a negative control. Bacteria were cultured overnight at 37°C in microtiter plates under static conditions. Reduction of *hmw1A* expression and inactivation of the *hmw1A* and *hmw1C* genes increased biofilm growth, compared to the rRdS WT strain (*, *P* < 0.05; **, *P* < 0.0005). (B) Reduction of *hmw1A* expression (strains P653 and P654) related to increased biofilm growth, compared to the P651 and P652 isolates (*, *P* < 0.0001). Representative images are shown; results of at least three independent experiments (*n* ≥ 3) in triplicate are shown as the mean OD_570_/OD_600_ ratios ± SEM. Statistical comparisons of means were performed by one-way ANOVA and Sidak’s multiple-comparison test. (C) Confocal laser scanning microscopy showing representative images of P652 (left) and P653 (right) biofilms. (Top and middle) Maximum-intensity projection (top) and *xz* plane lateral projection (middle) of confocal z-stack images of total biofilm thickness. (Bottom) 3D visualizations of the same data sets using IMARIS software. (D) Atomic force microscopy images showing representative images of P652 (left) and P653 (right) biofilms. (Top) Error signal images; (bottom) representative topography. Gaps where the mica surface was exposed are marked with asterisks.

Previous evidence pointed to serum antibodies as a selective pressure against NTHi strains with high HMW expression levels, leading to the downregulation of HMW1 by phase-variable changes in the promoter (39). Whether a high expression level of HMW1 serves an early role in infection is unknown, but here, we found that *hmw1A* phase variation regulates the hyperinvasive phenotype of strains with a particular HMW1 allele. We reasoned that the loss of HMW1 expression and hyperinvasion could lead to a lifestyle switch to favor extracellular survival. Given that biofilm formation relates to the extracellular persistence of NTHi (8), we measured biofilm formation on polystyrene microtiter plates using crystal violet staining of 24-h static cultures of the rRdS strain set with various SSRs at *hmw1* and of the four serially collected CT 73 strains. By using the rRdS series, with 14, 20, 21, 22, 23, or 24 SSRs in the *hmw1A* promoter region, increased repeat numbers enhanced biofilm formation to levels comparable to those of the RdS negative control that lacks the *hmw1* locus; no significant differences were seen among rRdS clones with 20 to 24 repeats. Furthermore, inactivation of the *hmw1A* or *hmw1C* gene also increased biofilm formation compared to the rRdS wild-type (WT) strain (Fig. 6A). We also found a strong correlation between SSRs and biofilm formation within CT 73, where later isolates, P653 and P654, produced higher biofilm biomass than that the early isolates P651 and P652 (Fig. 6B). Biofilm measurements of isolates within the other CTs carrying HMW adhesins (but not the hyperinvasive allele [CTs 18, 3, and 44]) also showed a negative correlation between biofilm formation and phase-variable HMW expression (Fig. S5C), which may indicate a more general trade-off between HMW expression and biofilm formation not only dependent on having the hyperinvasive allele.

Finally, we compared the biofilm architectures of high-invasion P652 and low-invasion P653 by using scanning confocal and atomic force microscopy. Spatial distribution (two-dimensional [2D]) and three-dimensional (3D) biofilm images were obtained after superimposing each z-stack series. Low-biofilm strain P652 had a less compact biofilm with patchy surface coverage (average thickness of 33.58 ± 1.87 μm), whereas strain P653 produced a compact biofilm structure covering the entire area (average thickness of 46.85 ± 1.65 μm) (Fig. 6C). Topography analysis of the shape, structure, and surface roughness of bacteria found no significant differences between P652 and P653. However, gaps could be observed when imaging the P652 strain in most images since the biofilm formed was not uniform and did not fully cover the mica surface, unlike images of the P653 strain (Fig. 6D). These results support that SSR-mediated phase variation at the *hmw1* promoter regulates a switch between epithelial adherence/invasion and extracellular biofilm lifestyles.

## DISCUSSION

The well-characterized adhesive glycoproteins HMW1 and HMW2 are expressed by NTHi to mediate adherence to human respiratory epithelial cells, and they enhance the ability of NTHi to colonize the nasopharynx and oropharynx of rhesus macaques (33–35, 41, 50). We had previously found that a particular allele of HMW1A (here HMW1A_hyper_) conferred NTHi with highly elevated rates of intracellular invasion, in which self-aggregated clumps of bacteria were seen invading host cell endosomes (30). Here, we extend our investigations into this hyperinvasive allele using experiments with clinical isolates of distinct origins as well as genetically engineered laboratory strains to reach our main conclusions: (i) we showed that NTHi hyperinvasion depends on HMW1A expression and glycosylation by HMW1C, extending our observations to two cultured epithelial cell types; (ii) using time-lapse microscopy, we confirmed that NTHi bacteria with HMW1A_hyper_ invade airway epithelial cells as bacterial aggregates that can persist and survive at least 24 h; (iii) we showed that although the HMW1A_hyper_ allele is relatively rare, its binding domain and predicted glycosylation pattern are highly diverged from those of both the canonical HMW1 and HMW2 proteins; (iv) we demonstrated that heptameric repeat expansion downstream of the P2 promoter decreases HMW1_hyper_ expression; and (v) we identified the presence of HMW1_hyper_ in a longitudinally collected series of persistent isolates that revealed a phase-variable switch from hyperinvasiveness to biofilm formation concomitant with *hmw1* heptameric repeat expansion and decreased *hmw1* expression. These findings suggest a potential lifestyle switch during NTHi pathoadaptation toward biofilm formation, which is mediated by phase-variable decreased expression of the HMW1 adhesin.

The *hmw* loci were present in 43.5% of the longitudinal isolates in our collection, consistent with other independent strain sets (32). Clinical isolates lacking the two *hmw* loci instead encode the adhesin Hia at another locus (51). Notably, as expected, pairwise alignment of HMW binding domains found extremely high diversity within putative HMW1 and HMW2 groups (32), and the hyperinvasion-associated binding domains were the most diverged. We found only six sequenced genomes with highly similar HMW_hyper_ alleles, the four COPD isolates of CT 73 from Spain (strains P651 to P654) and two pediatric OM isolates from the United States, the closely related strain PittEE and the distantly related strain 86-028NP (see Table S3 in the supplemental material). Two particular features distinguished the HMW1_hyper_ variants in these strains from other HMW1 proteins: an exclusive amino acid signature in their binding domains and the absence of eight residues known to be glycosylated in canonical HMW1_R2846_. We attempted to model the HMW1 protein structure using homology and fold recognition methods, but the absence of solved structures with high sequence identity and coverage yielded no reliable outcome. However, all models predicted that the adhesin domain, like the propiece, will adopt a β-solenoid superhelix, as does the hemopexin binding domain of the two-partner secretion system HxuB/HxuA of H. influenzae (data not shown) (52). The observed distribution of HMW protein sequences, including the “swapping” of binding domains between loci, suggests that deeper sequence analysis of HMW variants could be of use for predictive purposes, e.g., screening genomes for hyperinvasive allele types.

Phase variation of the heptameric repeat upstream of HMW-encoding genes is known to affect HMW expression levels, with increasing copy numbers leading to decreased RNA and protein expression levels. Hyperinvasion conferred by HMW1_hyper_ is likewise controlled by phase variation in the upstream promoter region, and moreover, we found that the SSR number affects HMW expression from the P2 promoter only. Our reporter fusions and sequence analyses found that P1 was not functional under the conditions tested. The putative transcription start site at P1 may correspond to an mRNA processing site since previous primer extension analyses could not discriminate transcriptional starts from these (36). Whether such an mRNA processing site modulates HMW expression is unclear. Thus, SSR variation affects the length of the 5′ UTR, but whether this directly affects transcription, modifies mRNA stability, and/or impacts translation requires further investigations. Likewise, *hmw* expression also undergoes epigenetic regulation as part of the phase-variable ModA methyltransferase phasevarion (53, 54). An additional complexity level by considering not only *hmw* phase variation but also its epigenetic regulation is out of the scope of this study and will require further work.

SSR phase variation within the human host has been observed in the *hmw* and IgA protease *igaB2* loci from a large set of prospectively collected NTHi strains from COPD patients (27, 28) and also during experimental human nasopharyngeal colonization (although the *hmw* promoter regions were not specifically analyzed) (55). Also, a previous analysis of serial COPD isolates showed that HMW expression typically decreases over time, likely in response to selective pressure from the high titer of anti-HMW antibodies in COPD patient sera tested against purified HMW1 protein from strain R2846 (39). This is compatible with phase-variable genes also lowering expression during long-term persistent meningococcal carriage, in part due to antibody-mediated selection (56). The fact that high antibody titers are present even at the time of initial lower airway infection makes it unclear what advantage high levels of HMW may confer at early stages of infection. One possibility could relate to (at least for some alleles) HMW1 showing preferential binding to 2-3-linked sialic acid glycans that are predominant in the lower respiratory tract (57). Here, we show a phase-variable decrease of HMW levels over time in independent sets of serial COPD isolates, supporting this as being common during NTHi persistence within the COPD lung environment.

Shifting from a planktonic lifestyle to a biofilm community has been observed for several chronic bacterial infections (58). For example, within the COPD lung, Pseudomonas aeruginosa evolves toward increased mutation rates and antibiotic resistance, reduced production of proteases and motility, and production of biofilms (59). We speculated that phase-variable downregulation of HMW1A_hyper_ might be coupled to a shift from one chronicity-associated phenotype (intracellular invasion) to another (biofilm formation), both of which could allow NTHi to persist, for example, by increasing the survival of genetically sensitive bacteria during antibiotic treatment. Phase-variable expression of HMW1 was not only correlated with increased hyperinvasiveness when the HMW1_hyper_ allele was present but also inversely correlated with biofilm formation. This negative correlation with biofilm levels held both for laboratory-engineered strains in which the *hmw1*_hyper_ locus was isolated in a distinct strain background (rRdS) and in multiple clinical isolate sets collected from COPD patients. These data suggest that phase-variable decreased HMW expression over time during persistent lung colonization, likely by antibody selective pressure, leads to a switch in NTHi’s lifestyle from high adherence/invasiveness to higher biofilm formation. These data further suggest that HMW expression may generally inhibit biofilm formation. Although we originally speculated that the self-aggregation of high-expressing HMW1A_hyper_ strains could be responsible for the patchiness of their biofilms, the inverse correlation was not restricted to HMW1A_hyper_ strains. Instead, HMW expression in general may favor host cell interactions over biofilm formation.

We had previously identified recurrent loss-of-function mutations in the NTHi *ompP1* (*fadL*) gene using the same set of longitudinally collected isolates (4). These mutations led to decreased adhesion/invasion but also gave resistance to arachidonic acid, which is abundant in COPD lungs. We speculated that the pathoadaptive loss of *ompP1* might restrict these strains to the COPD lung environment since nearly all nonlung isolates had an intact gene. Here, in contrast, the phase-variable loss of HMW1 adhesin expression is more readily reversible, allowing for a future switch back to high HMW expression and low biofilm formation through contraction of the heptamer repeat.

**FIG 7:**
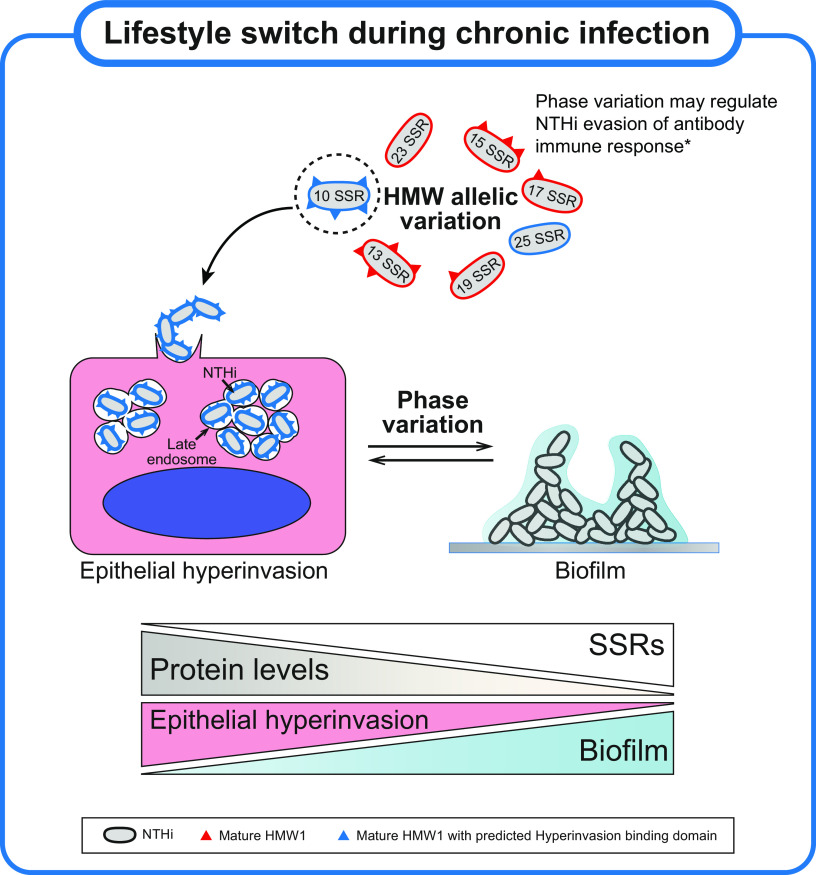
Model illustrating HMW SSR phase variation and its potential regulation of the H. influenzae lifestyle during persistence. The HMW adhesin, whose expression is regulated by phase variation consisting of changes in the number of 7-bp tandem repeats in its promoter region, binds to host cell receptor(s) through its highly variable binding domain. We provide evidence for phase variation likely regulating a bacterial lifestyle switch between invasion-subcellular location, as well as extracellular biofilm growth during NTHi persistence. Besides identifying shared features in the binding domains of HMW variants associated with epithelial hyperinvasion (HMW1 allelic variation), reduced *hmw1A* expression and HMW1A protein levels due to increased (SSR)*_n_* lower NTHi’s ability to hyperinvade epithelia but also increase its ability to form biofilms. HMW-mediated cell infection may be essential for virulence at early stages of infection, but its persistence may indeed be favored by limiting HMW to thus not only overcome antibody selective pressure (*, previously suggested by Cholon and coauthors [39]) but also find a balance between amenable lifestyles favoring chronicity.

In conclusion, HMW-mediated hyperinvasion is associated with specific *hmw1A* allelic variants, which may facilitate early stages of airway infection. However, HMW expression may often be downregulated by phase variation over time, driving a phenotypic switch from bacteria living as intracellular groups to an extracellular biofilm lifestyle. This could serve as an adaptive strategy during NTHi persistence (Fig. 7). The mutability of SSRs at phase-variable loci is determined by a combination of environmental, population, and molecular drivers that will affect the evolution of these tracts (60). A further understanding of which and how drivers affect the mutability of the tandem repeats in the *hmw* promoter regions will shed light on the drivers of these reversible pathoadaptations.

## MATERIALS AND METHODS

The generation of bacterial strains and plasmids; bacterial growth on agar plates and liquid cultures; bacterial biofilm formation, monitored by crystal violet staining, confocal microscopy, and atomic force microscopy; cultured cell procedures involving bacterial infection, live imaging, and immunofluorescence microscopy on fixed samples; and gene expression and protein immunodetection procedures are detailed in Text S1 in the supplemental material.

10.1128/mBio.00789-21.1 TEXT S1 Additional detailed experimental methods. Download Text S1, DOCX file, 0.06 MB. Copyright © 2021 Fernández-Calvet et al. 2021 Fernández-Calvet et al. https://creativecommons.org/licenses/by/4.0/ This content is distributed under the terms of the Creative Commons Attribution 4.0 International license.

### Bacterial strains, plasmids, media, and growth conditions.

Strains and plasmids used in this study are listed in Table 1 and Table S1, respectively. Clinical strains belong to a genome-sequenced longitudinal collection recovered from respiratory samples of COPD patients (BioProject accession number PRJNA282520) (4). NTHi strains were grown at 37°C with 5% CO_2_ on chocolate agar PolyViteX (PVX; bioMérieux) or Haemophilus test medium (HTM) base agar (Oxoid) supplemented with 10 μg/ml hemin and 10 μg/ml NAD, referred to sHTM agar. NTHi liquid cultures were grown at 37°C with 5% CO_2_ in supplemented brain heart infusion (sBHI) medium. Erythromycin at 11 μg/ml (Erm_11_), kanamycin at 30 μg/ml (Km_30_), or spectinomycin at 30 μg/ml (Spec_30_) was used when required. Escherichia coli was grown on Luria-Bertani (LB) medium or LB agar at 37°C, with ampicillin at 100 μg/ml (Amp_100_), chloramphenicol at 20 μg/ml (Cm_20_), erythromycin at 150 μg/ml (Erm_150_), or kanamycin at 50 μg/ml (Km_50_), when necessary.

10.1128/mBio.00789-21.7 TABLE S1 Plasmids used in this study. Download Table S1, DOCX file, 0.1 MB. Copyright © 2021 Fernández-Calvet et al. 2021 Fernández-Calvet et al. https://creativecommons.org/licenses/by/4.0/ This content is distributed under the terms of the Creative Commons Attribution 4.0 International license.

### Protein sequence alignments and molecular modeling.

To perform HMW1/2 assignments, the binding domains defined by R2846 sequences as amino acids 555 to 914 in HMW1A and amino acids 553 to 916 in HMW2A were used to calculate the percent amino acid identities of the 12 HMW binding domains of the six isolates with fully assembled genomes using Clustal Omega (Table 2). Homology models of the HMW1 propiece [(HMW1-PP)_NTHi_] from strains 86-028NP, PittEE, and P652 (representative of CT 73) were built automatically using the SWISS-MODEL server (61), which previously identified the crystal structure of HMW1-PP_R2846_ from NTHi strain R2846 (1.92-Å resolution; PDB accession number 2ODL) (46) as the best possible template (100% coverage; 95.43 to 97.04% identity). Structural and energetic evaluations of the models and template compared well. Structure visualization and figure preparation were performed with Edu PyMOL version 1.7.4 software.

### SNP-based phylogeny.

The genomic relationship between strains 86-028NP, R2846, PittEE, and P652 (used as a reference genome) was analyzed with CSI Phylogeny version 1.4 (62) according to single nucleotide polymorphisms (SNPs) between core genomes (92.2% of the reference genome covered by all strains). The minimum distance between SNPs was set at 10 bp. For visualization and figure preparation, the iTOL online tool was used (https://itol.embl.de/).

### Statistical analysis.

In all cases, a *P* value of <0.05 was considered statistically significant. Analyses were performed using the Prism software, version 7, statistical package for Mac (GraphPad Software). Each analysis and its corresponding results are detailed in each figure legend.
