# Supplementary figures and images for: Phase Variation in HMW1A Controls a Phenotypic Switch in Haemophilus influenzae Associated with Pathoadaptation during Persistent Infection
Source: mBio. 2021 Jun 22;12(3):e00789-21. doi: 10.1128/mBio.00789-21 (PMC8262952; doi:10.1128/mBio.00789-21)

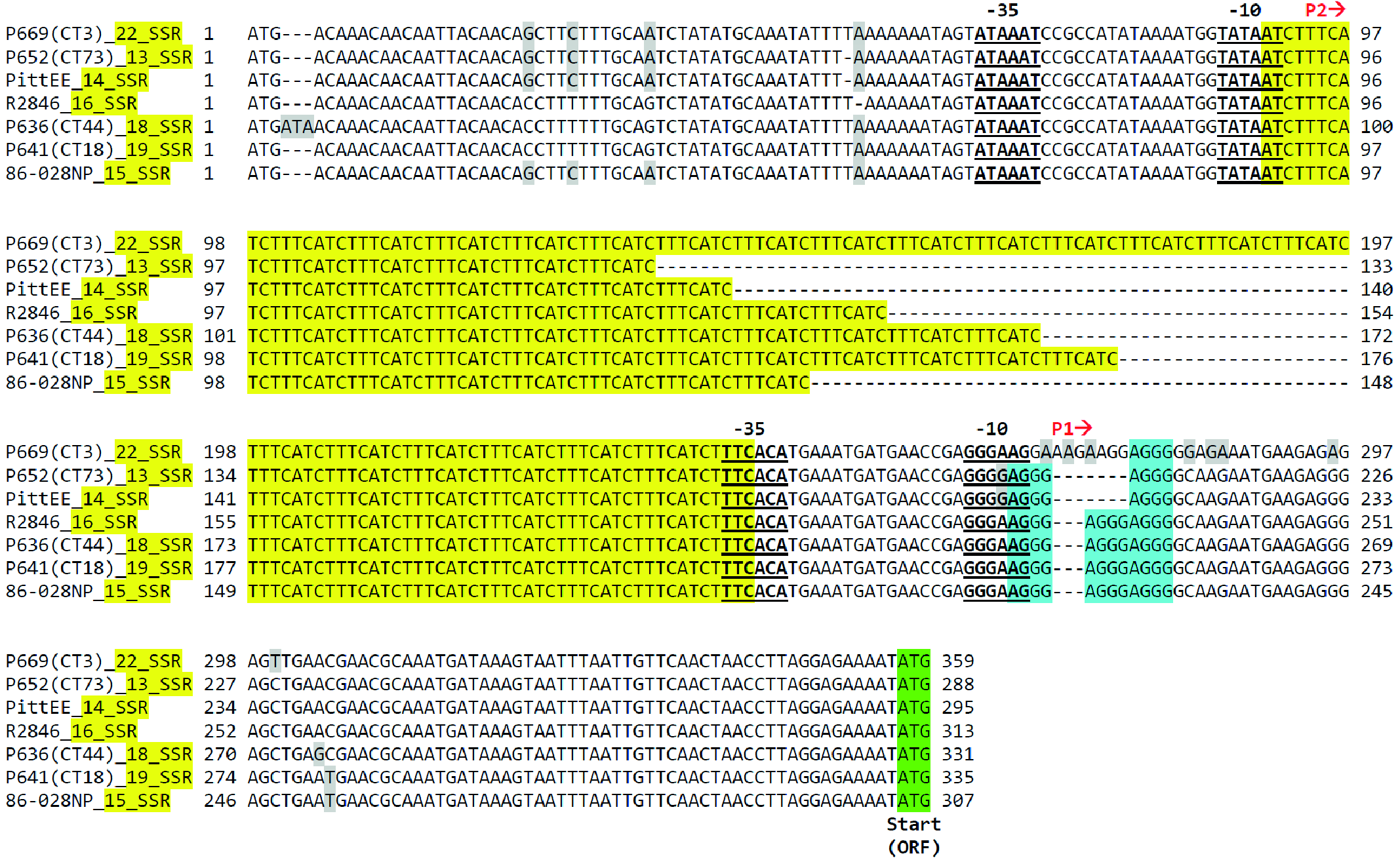

Supplement: FIG S2 [file mbio.00789-21-sf002.tif]

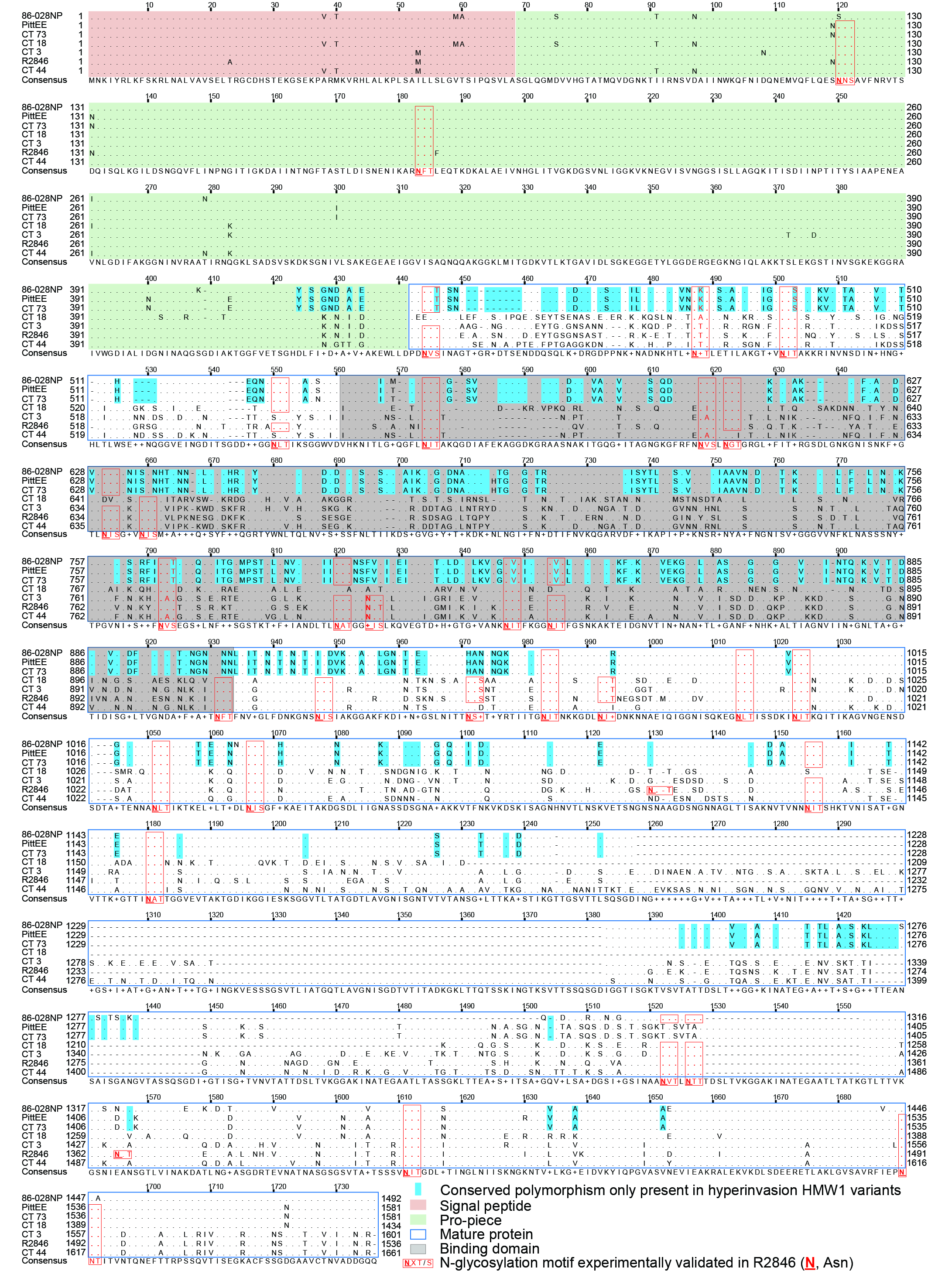

Supplement: FIG S3 [file mbio.00789-21-sf003.tif]

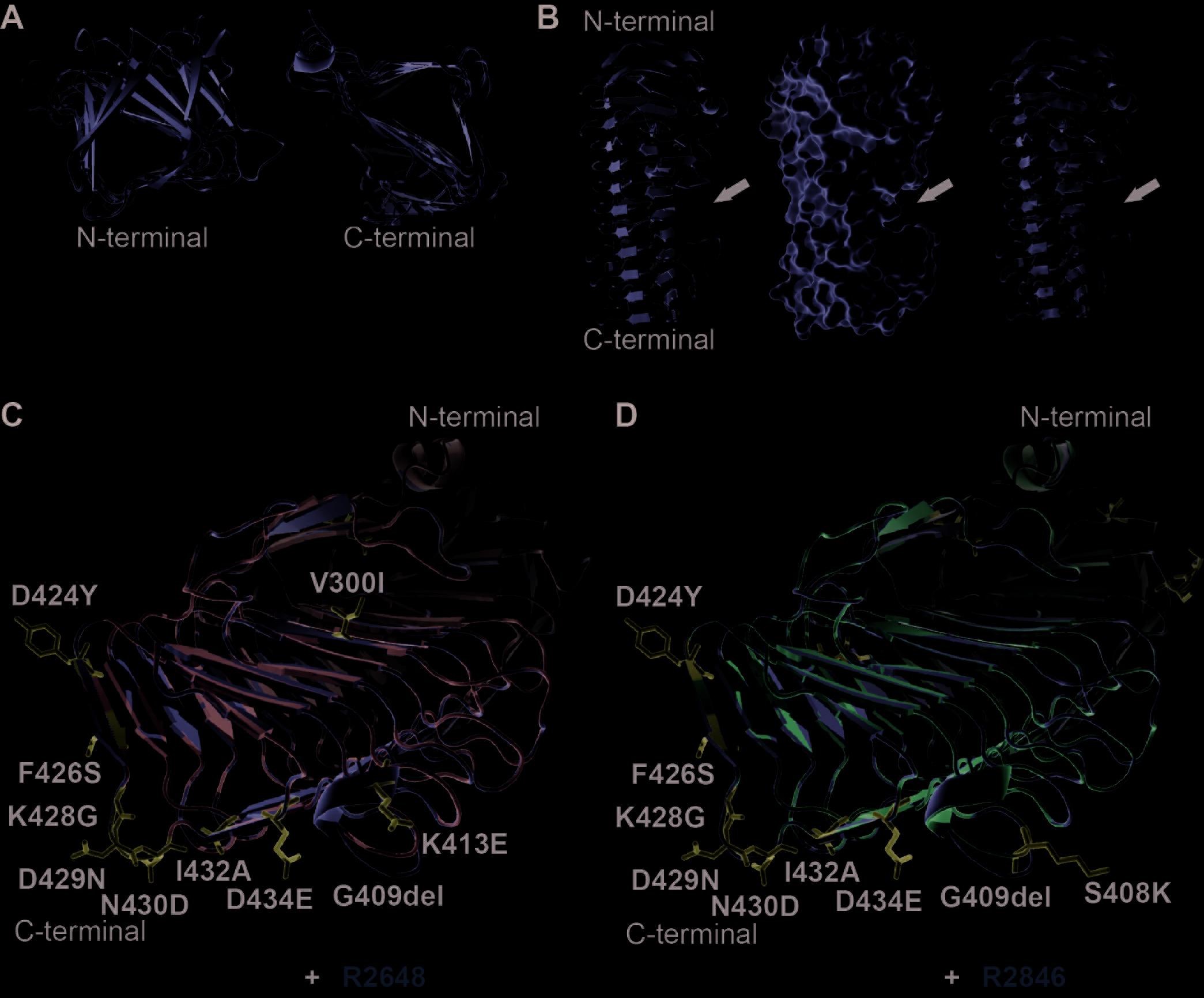

Supplement: FIG S4 [file mbio.00789-21-sf004.tif]

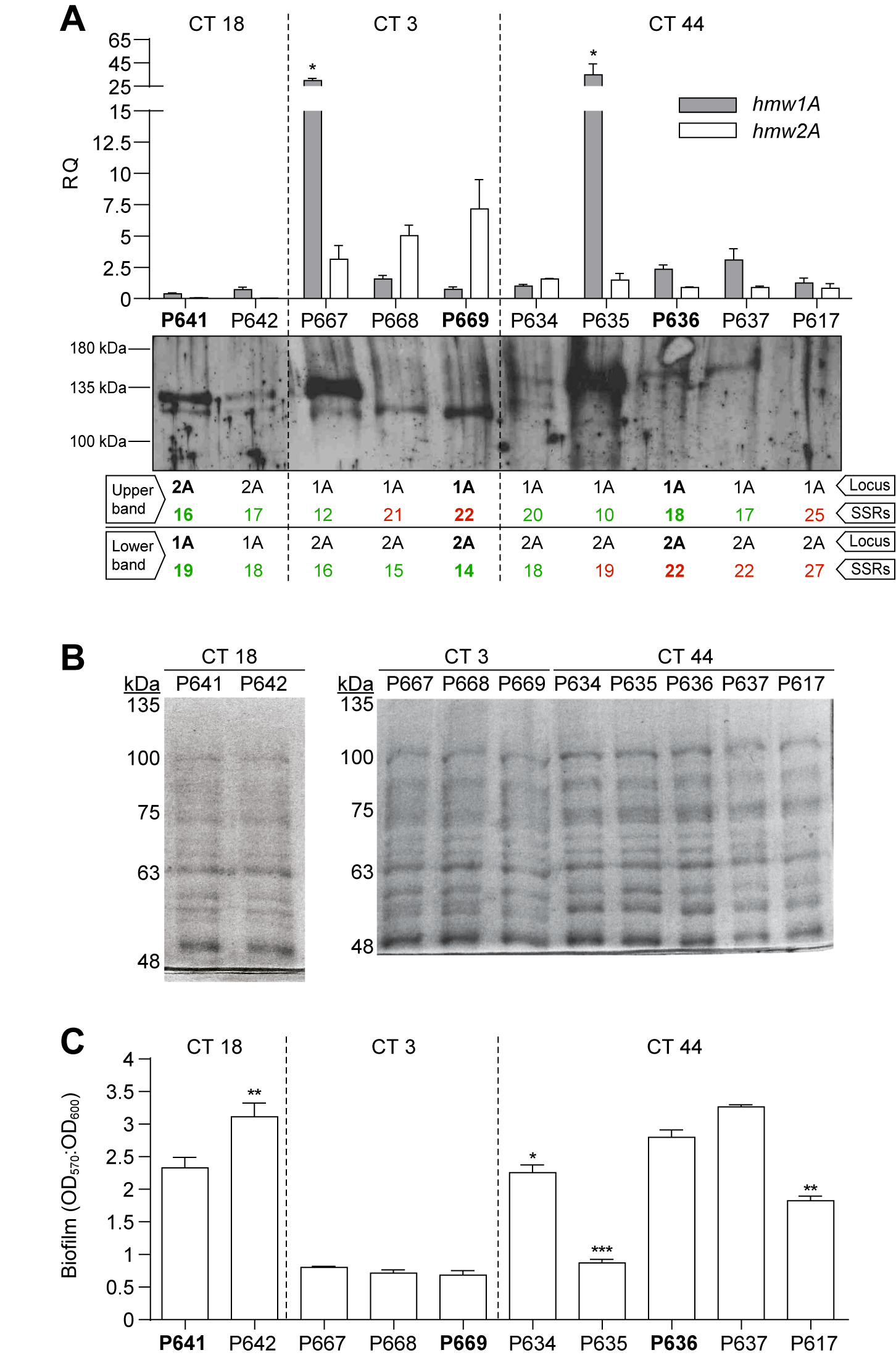

Supplement: FIG S5 [file mbio.00789-21-sf005.tif]
