# Supplementary material for: Phase Variation in HMW1A Controls a Phenotypic Switch in Haemophilus influenzae Associated with Pathoadaptation during Persistent Infection
Source: mBio. 2021 Jun 22;12(3):e00789-21. doi: 10.1128/mBio.00789-21 (PMC8262952; doi:10.1128/mBio.00789-21)
Supplement: TABLE S2 [file mbio.00789-21-st002.docx]

**Table S2.** Primers used in this study.

| **Primer (name/#ID)** | **Primer sequence (5’-3’)** | **Purpose** |
| --- | --- | --- |
| Pr−hmw1A−22rep−KpnI−F2/#1918 | CGGGGTACCTAACTCATTCGTCAGTGTTA | Sanger sequencing of *hmwA* promoter (*yrbI*-proximal); PCR |
| Prhmw1A(NP)−BamHI−R/#1629 | CGCGGATCCTATTTTCTCCTAAGGTTAGTTG |  |
| hmwA−radA(P652)/#1656 | ATAGGTGTTGCCCAAAAATAA | Sanger sequencing of *hmwA* promoter (*radA*-proximal) |
| Prhmw1A(NP)−BamHI−R/#1629 | CGCGGATCCTATTTTCTCCTAAGGTTAGTTG |  |
| hmw1A−7−D/#13 | CACTCGCTAGTGGTTGTTAATGATGAA | PCR |
| hmw1A−recomb−strat2/#1273 | TATCCCCCAACTTTAATCGCGTCT |  |
| hmw1A−InvPCR−F/#1275 | ATTGTATGGGGCGATATTGCGTTA | Inverse PCR |
| hmw1A−InvPCR−R/#1276 | ATGTGACTTTATCACCTGTAATCA |  |
| HMW1B(NP)−F/#1817 | TTAGGTTTTGTTAATGCCAATTT | PCR |
| HI1680(Rd)−R/#1820 | CAAATTCATACGGAACAAATCA |  |
| HMW1B(NP)−XhoI−R/#1818 | CCGCTCGAGTTAGAAACTGAATGTTAATC' | Inverse PCR |
| HI1680(Rd)+NcoI−F/#1819 | CATGCCATGGACTTTTATATTTATAACGCTC |  |
| ermC−xhoI−F/#1517 | CCGCTCGAGGAGCTCGAATTCGGCTTCATGCT | PCR |
| ermC−NcoI−R/#1518 | CATGCCATGGGGTACCGAGCTCGAATTCGGCTTGATCG |  |
| NTHI1981-F1/#1916 | ATGCCTAATTTTTCATTCGTA |  |
| yrbI-KpnI-R2/#1917 | CGGGGTACCTTATTGCCCCATATTTTTCA |  |
| hmw1A(NP) −R/#1919 | TATTATATCGCTGATGGTGA |  |
| qPCRhmwA−F2/#1979 | GACGCCAAAGAGTGGTTGTT | qPCR *hmw1A*  (strains P641 and P642) |
| qPCRhmwA−R2/#1980 | TCAAGCGTTGCGTTTGTTAG |  |
| qPCRhmwA−F4/#1983 | AACGCTGTCGCTAAAACAAAA | qPCR *hmw2A*  (strains P641 and P642) |
| qPCRhmwA−R4/#1984 | GCACCATTATCAGTGCGAGA |  |
| qPCRhmwA−F5/#1985 | GAGACATCGGGGCATTATTT | qPCR *hmw2A* (strains P617, P634, P635, P636 and P637) |
| qPCRhmwA−R5/#1986 | TACCGCGAGAAGAGGAAGAG |  |
| qPCRhmwA−F6/#1987 | CCATTAACGCAGGCACATC | qPCR *hmw1A* (strains P617, P634, P635, P636 and P637) |
| qPCRhmwA−R6/#1988 | TGGGGTTATCCTTTCCTCCT |  |
| qPCRhmwA−F9/#1993 | GTGATTCCGTGGCTTTTGAG | qPCR *hmw2A* (strains P667, P668, P669) |
| qPCRhmwA−R9/#1994 | ACCCTGTGCGGTAATTTGAG |  |
| qPCRhmwA−F2/#1979 | GACGCCAAAGAGTGGTTGTT | qPCR *hmw1A* (strains P667, P668, P669) |
| qPCRhmwA−R10/#1995 | TGTCGGCTTGTCTTGTTTGT |  |
| qPCRhmw1A−F/#1649 | TGTATCACTAAATGGAACTGGTAGAGG | qPCR *hmw1A* (strains P651, P652, P653, P654, 86-028NP, rRdS, rRdS derivative mutants) |
| qPCRhmw1A−F/#1650 | CCCTGGCGATGAAAATAAGA |  |
| qPCRhmw2A−F/#1651 | ATCCCATGTTCGCAAGGATA | qPCR *hmw2A* (strains P651, P652, P653, P654) |
| qPCRhmw2A−F/#1652 | AATTTTGCCCACCAAGAGTG |  |
| qPCRhmw2A−F/#1682 | TCTGTTTTAGCGAGCGGTTT | qPCR *hmw2A* (strains 86-028NP, rRdS, rRdS derivative mutants) |
| qPCRhmw2A−F/#1683 | GAATACGGCGGAGTTGTTGT |  |
| gyrA−F2/#1078 | ATATGTTGGTTGATGGGCAAGG | qPCR *gyrA* (endogenous control, present in all strains) |
| gyrA−R2/#1079 | GGCGAGAAATTGACGGTTTCT |  |
| EUB338 | GCTGCCTCCCGTAGGAGT | FISH |
| GAM42a | GCCTTCCCACATCGTTT |  |
| P-hmw1A_Fw_SphI_v2/#2082 | ACATGCATGCTAACTCATTCGTCAGTGTTATTG | PCR |
| P-hmw1A_Rv_EcoRI/#2083 | CCGGAATTCAACAATTAAATTACTTTATCATTTGCGT |  |
| Erm_Fw_NarI | GCATGCAAGGTTTCCTAAAATCAGTGAAAA |  |
| Erm_Rv_XhoI_BglII | ACTAGTCATATGTATATCTCCTTCTTAAATCTGAATTCAACAATTAAATTACTTTATCATTTGCGT |  |
| Seq_primer_univ_AT_NheI | GCTAGCCGTATTACCGCCTTTGAGTG |  |
| pCN_univ_rv_AT | GTTTTGGTTCATCTTCTGTTAACTTACTAA |  |
| Pr-Hmw1A-Fw_SphI | GCATGCAAGGTTTCCTAAAATCAGTGAAAA |  |
| Pr-Hmw1A_Rv-EcoRI_RBScons_SpeI | ACTAGTCATATGTATATCTCCTTCTTAAATCTGAATTCAACAATTAAATTACTTTATCATTTGCGT |  |
| Rep_Rv_EcoRI/#2084 | CCTCGGTTCATCATTTCATGTGAATTCGAAAGATGAAAGATG |  |
| Rep_Fw_SphI/#2085 | AATCCGCCATATAAAATGGTATAGCATGCATCTTTCATCTTTC |  |
| P1_Hmw1A_SphI/#2086 | ACATGCATGCATCTTTCATCTTTCACATGAAATGATGAACCGA |  |
| P2_Hmw1A_EcoRI/#2087 | CCGGAATTCGAAAGATGAAAGATTATACCATTTTATATGGCGGA  TT |  |
| LB74/#2088 | AAGTGTTGGCCATGGAACAG | Sanger sequencing of *P_hmw_::gfp* transcriptional reporter plasmids |
| pTBH-seq-fw/#2089 | CTGACACCCTCATCAGTGC | Sanger sequencing of *P_hmw_::gfp* transcriptional reporter plasmids |
