# Supplementary material for: Phase Variation in HMW1A Controls a Phenotypic Switch in Haemophilus influenzae Associated with Pathoadaptation during Persistent Infection
Source: mBio. 2021 Jun 22;12(3):e00789-21. doi: 10.1128/mBio.00789-21 (PMC8262952; doi:10.1128/mBio.00789-21)
Supplement: TABLE S3 [file mbio.00789-21-st003.docx]

**Table S3.** tBLASTn against published *H. influenzae* genomes using the HMW1_86-028NP_ binding domain (residues 542-908) as query sequence. Results filtered by query cover (>90%). First 25 matching results are shown (search date, March 10^th^, 2021).

| ***H. influenzae* strains** | **Query cover** | **E value** | **Identity** | **Accession** | **Locus tag** |
| --- | --- | --- | --- | --- | --- |
| 86-028NP | 96% | 0 | 100.00% | CP000057.2 | NTHI1983 |
| A950006 | 96% | 0 | 99.72% | AF180944.1 | AAD56660.1 |
| PittEE | 96% | 0 | 99.44% | CP000671.1 | CGSHiEE_03650 |
| P652 | 96% | 0 | 99.44% | CP031684.1 | CH613_07605 |
| I213 | 96% | 0 | 88.42% | KJ859655.1 | KJ859655.1 |
| C486 | 96% | 0 | 88.42% | CP007471.1 | NTHIC486_01112 |
| F894 | 96% | 6e-92 | 49.44% | KJ859625.1 | KJ859625.1 |
| F286-5 | 96% | 2e-91 | 49.17% | KJ859637.1 | KJ859637.1 |
| 48P153H1 | 96% | 4e-91 | 49.17% | CP020007.1 | BV085_1785 |
| 6P32H1 | 96% | 4e-91 | 49.17% | CP020013.1 | BV140_1469 |
| 6P32H2 | 96% | 4e-91 | 49.17% | CP020012.1 | BV139_1466 |
| 48P106H1 | 96% | 2e-90 | 48.89% | CP020006.1 | BV083_1787 |
| I198 | 96% | 7e-90 | 48.31% | KJ859649.1 | KJ859649.1 |
| F441 | 96% | 3e-89 | 48.34% | KJ859622.1 | KJ859622.1 |
| I202 | 96% | 1e-88 | 48.03% | KJ859651.1 | KJ859651.1 |
| 477 | 96% | 3e-86 | 45.38% | CP007470.1 | NTHI477_00062 |
| F1296-5 | 96% | 6e-85 | 46.26% | KJ859630.1 | KJ859630.1 |
| K19RE | 96% | 4e-84 | 45.98% | KJ859673.1 | KJ859673.1 |
| F1942 | 96% | 1e-78 | 45.13% | KJ859635.1 | KJ859635.1 |
| F164-6 | 96% | 3e-78 | 44.85% | KJ859632.1 | KJ859632.1 |
| 723 | 96% | 5e-78 | 44.92% | CP007472.1 | NTHI723_01789 |
| I336 | 93% | 7e-78 | 45.66% | KJ859667.1 | KJ859667.1 |
| G922 | 96% | 7e-77 | 46.45% | KJ859644.1 | KJ859644.1 |
| F1015 | 96% | 8e-77 | 46.47% | KJ859628.1 | KJ859628.1 |
| F1015 | 96% | 8e-77 | 46.47% | KJ859629.1 | KJ859629.1 |
