# Supplementary material for: Phase Variation in HMW1A Controls a Phenotypic Switch in Haemophilus influenzae Associated with Pathoadaptation during Persistent Infection
Source: mBio. 2021 Jun 22;12(3):e00789-21. doi: 10.1128/mBio.00789-21 (PMC8262952; doi:10.1128/mBio.00789-21)
Supplement: TEXT S1 [file mbio.00789-21-t0001.docx]

**Supplementary Information Text for:**

**Phase variation in HMW1A controls a phenotypic switch in *Haemophilus influenzae* associated with pathoadaptation during persistent infection**

Ariadna Fernández-Calvet^1^, Begoña Euba^1^, Celia Gil-Campillo^1^, Arancha Catalan-Moreno^1^, Javier Moleres^1^, Sara Martí^2-3^, Alexandra Merlos^4^, Jeroen D. Langereis^5^, Francisco García-del Portillo^6^, Lauren O. Bakaletz^7^, Garth D. Ehrlich^8-12^, Eric A. Porsch^13^, Margarita Menéndez^2,14^, Joshua Chang Mell^8-11^, Alejandro Toledo-Arana^1^ and Junkal Garmendia^1,2#^

^1^Instituto de Agrobiotecnología, Consejo Superior de Investigaciones Científicas (IdAB-CSIC)-Gobierno de Navarra, Mutilva, Spain;

^2^Centro de Investigación Biomédica en Red de Enfermedades Respiratorias (CIBERES), Madrid, Spain;

^3^Microbiology Department, Hospital Universitari Bellvitge, University of Barcelona, IDIBELL, Barcelona, Spain;

^4^Department of Pathology & Experimental Therapeutics, IDIBELL-University of Barcelona, L’Hospitalet de Llobregat, Spain;

^5^Section Pediatric Infectious Diseases, Laboratory of Medical Immunology, Radboud Institute for Molecular Life Sciences and Radboud Center for Infectious diseases, Radboudumc, Nijmegen, The Netherlands;

^6^Laboratorio de Patógenos Bacterianos Intracelulares, Centro Nacional de Biotecnología (CNB)-CSIC, Madrid, Spain;

^7^Center for Microbial Pathogenesis, The Research Institute at Nationwide Children’s Hospital and The Ohio State University College of Medicine, Columbus, Ohio, USA;

^8^Department of Microbiology and Immunology, Drexel University College of Medicine, Philadelphia, Pennsylvania, USA;

^9^Center for Genomic Sciences, Drexel University College of Medicine, Philadelphia, Pennsylvania, USA;

^10^Center for Advanced Microbial Processing, Drexel University College of Medicine, Philadelphia, Pennsylvania, USA;

^11^Center for Surgical Infections and Biofilms, Institute for Molecular Medicine and Infectious Disease, Drexel University College of Medicine, Philadelphia, Pennsylvania, USA;

^12^Department of Otolaryngology, Head and Neck Surgery, Drexel University College of Medicine, Philadelphia, Pennsylvania, USA;

^13^Department of Pediatrics, The Children's Hospital of Philadelphia, Philadelphia, Pennsylvania, USA;

^14^Instituto de Química Física Rocasolano (IQFR-CSIC), Madrid, Spain

#Address correspondence to:

Junkal Garmendia

Email: [juncal.garmendia@unavarra.es](mailto:juncal.garmendia@unavarra.es); [juncal.garmendia@csic.es](mailto:juncal.garmendia@csic.es)

**Supplemental Methods and Materials**

**Generation of *H. influenzae* rRdS derivative mutant strains.** Primers used in this study are listed in **Table S2**. As a surrogate strain for genetic manipulation to remove confounding issues with the paralogous *hmw2* operon, we used rRdS, a RdKW20 derivative strain which acquired the *hmw1ABC*_86-028NP_ operon by natural transformation (1). *Phusion* DNA Polymerase (Thermofisher Scientific) was used for PCR amplifications. To disrupt *hmw1A*, a 3,093 bp DNA fragment corresponding to the full open-reading frame (2,048 bp) plus 1,045 bp upstream was PCR amplified from 86-028NP genomic DNA with primers hmw1A-7-D and hmw1A-recomb-strat2. This PCR product was cloned into pGEM-T easy (Promega), generating pGEM-T-10 c.2, which was then linearized by inverse PCR using primers hmw1A-InvPCR-F and hmw1A-InvPCR-R to disrupt the *hmw1A* sequence, followed by ligation to a blunt-ended Erm resistance cassette obtained from pBSLerm *Sma*I digestion (2). This generated pGEM-T-Δ*hmw1A::ermC*. The *hmw1A::ermC* disruption cassette (4,114 bp) was PCR amplified with primers hmw1A-7-D and hmw1A-recomb-strat2 from pGEM-T-Δ*hmw1A::ermC*, and this amplicon was used for rRdS natural transformation using the MIV method (3). rRdSΔ*hmw1A::ermC* mutants were selected on sBHI agar with Erm_11_ and confirmed by PCR (primers hmw1A-7-D and hmw1A-recomb-strat2).

To disrupt *hmw1C*, a 4,336 DNA fragment containing the *hmw1C* gene (1,953 bp) and its flanking regions (1,202 bp upstream and 1,179 bp downstream) was PCR amplified from rRdS genomic DNA with primers HMW1B(NP)-F and HI1680(Rd)-R, followed by cloning into pJET1.2/blunt (Thermofisher Scientific) to generate pJET/*hmw1BC*-HI1680. This plasmid was linearized by inverse PCR with primers HMW1B(NP)+XhoI-R and HI1680(Rd)+NcoI-F, to delete a 2,224 bp DNA fragment corresponding to the *hmw1C* gene and its flanking regions (194 bp-upstream and 77 bp-downstream). In parallel, an Erm resistance cassette was PCR amplified with primers ermC-XhoI-F and ermC-NcoI-R using pBSLerm as a template. These PCR products were double-digested with *Xho*I and *Nco*I and ligated to generate pJET/Δ*hmw1C::ermC*. The *hmw1C::ermC* disruption cassette (3,296 bp) was PCR amplified from pJET/Δ*hmw1C::ermC* using primers HMW1B(NP)-F and HI1680(Rd)-R to produce the final construct used for rRdS natural transformation. rRdSΔ*hmw1C::ermC* mutants were selected on sBHI agar with Erm_11_ and confirmed by PCR (primers HMW1B(NP)-F and HI1680(Rd)-R).

To generate distinct SSR copy number at *hmw1A*_86-028NP_ promoter region for investigating the consequences of phase variation, a 1,200 bp DNA fragment containing the *hmw1A*_86-028NP_ promoter region with 22 (5´-ATCTTTC) tandem repeats (465 bp) and the 5´ end of the *hmw1A*_86-028NP_ gene (735 bp) was commercially synthesized (GeneArt Gene Synthesis, Invitrogen). This DNA fragment was PCR amplified with primers Pr-hmw1A-22rep-KpnI-F2 and hmw1A(NP)-R using pMK-Pr-*hmw1A*-22rep plasmid as template. In parallel, a 1,566 bp DNA fragment upstream the *hmw1A*_86-028NP_ promoter region, was PCR amplified with primers NTHI1981-F1 and yrbI-KpnI-R2. Both PCR products were *Kpn*I-digested and ligated. The ligation product was amplified with primers NTHI1981-F1 and hmw1A(NP)-R to generate a 2,766 bp product, which was then ligated into pJET1.2/blunt to generate pJET/NTHI1981-22rep-*hmw1A*. This plasmid was linearized by *Kpn*I digestion and ligated to an Erm resistance cassette obtained by pBSLerm *Kpn*I digestion, to generate pJET/NTHI1981-*ermC*-22rep-*hmw1A*. Finally, a 3,954 bp promoter replacement cassette was generated by amplification from this plasmid with primers NTHI1981-F1 and hmw1A(NP)-R, followed by natural transformation into rRdS. Transformants were selected on sBHI agar with Erm_11_ and confirmed by PCR (primers NTHI1981-F1 and hmw1A(NP)-R). Twenty independent clones were stored, and their *hmw1A* promoter sequences were Sanger sequenced, generating a panel of independent rRdS derivative clones with a variable number of tandem repeats, from 20 to 24 SSRs (rRdS-20 to rRdS-24), expected to reduce transcriptional activity from the *hmw1* locus with increasing repeats.

**Construction of *P_hmw_::gfp* transcriptional reporter plasmids.** First, a backbone plasmid suitable for maintenance in *H. influenzae* was generated to include an Erm resistance cassette and a multiple cloning site (MCS). The Erm resistance cassette (ErmC) was PCR amplified using pBSLerm as template and primers Erm_Fw_NarI and Erm_Rv_XhoI_BglII. The MCS was PCR amplified from the pCN47 plasmid (4) using primers Seq_primer_univ_AT_NheI and pCN_univ_rv_AT. PCRs products were separately cloned into pJET1.2/blunt. MCS and ErmC DNA fragments were excised using *Nhe*I/*Nar*I and *Nar*I/*Bgl*II, respectively. Both fragments were simultaneously ligated into *Nhe*I/*BamH*I digested pACYC177 (New England Biolabs), generating the pTBH-01 plasmid. Second, the *hmw1A* promoter region (P*_hmw_*) was PCR amplified by using 86-028NP genomic DNA as template and primers Pr-Hmw1A-Fw_SphI and Pr-Hmw1A_Rv-EcoRI_RBScons_SpeI, and cloned into pJET1.2/blunt. This *P_hmw_* fragment (*Sph*I/*Spe*I digested), and the ATG-less green fluorescent protein (GFP) module (*Spe*I/*Nar*I digested from the pHRG plasmid (5)) were simultaneously ligated into *Sph*I/*Nar*I digested pTBH-01, generating the pTBH03-*P_hmw_* reporter plasmid. Plasmids carrying the *P_hmw_* promoter variants were generated by PCR amplification of the different *P_hmw_* promoter regions with appropriate primer pairs (for details, see **Tables S1** and **S2**), cloned into pJET1.2/blunt, generating pJET1.2-P2-SSR_13_-P1, pJET1.2-P2-SSR_24_-P1, pJET1.2-P2-SSR_14_, pJET1.2-P2-SSR_24_, pJET1.2-SSR_14_-P1, pJET1.2-SSR_24_-P1, and confirmed by sequencing. Each corresponding DNA fragment was excised by *Sph*I/*EcoR*I digestion, followed by ligation into *Sph*I/*EcoR*I-digested pTBH03-*P_hmw_*, generating pTBH03-P2-SSR_13_-P1, pTBH03-P2-SSR_24_-P1, pTBH03-P2-SSR_14_, pTBH03-P2-SSR_24_, pTBH03-SSR_14_-P1 and pTBH03-SSR_24_-P1. pTBH03-P2-(SSR)_13_-P1 was used as a template for inverse PCR with primers P2_Hmw1A_EcoRI and P1_Hmw1A_SphI. PCRs products were independently digested with *EcoR*I or *Sph*I and religated, generating pTBH03-P2 and pTBH03-P1, respectively. All plasmids and SSR counts were confirmed by Sanger sequencing. This set of plasmids was independently introduced into *H. influenzae* RdKW20 by electroporation. Transformants were selected on sHTM agar with Erm_11_, generating the strain set: RdKW20 pTBH03-P2-SSR_13_-P1, RdKW20 pTBH03-P2-SSR_23_-P1, RdKW20 pTBH03-P2, RdKW20 pTBH03-P1, RdKW20 pTBH03-P2-SSR_14_, RdKW20 pTBH03-P2-SSR_24_, RdKW20 pTBH03-SSR_13_-P1 and RdKW20 pTBH03-SSR_24_-P1. All constructs and SSR count were re-confirmed after transformation into the RdKW20 strain background by plasmid extraction and Sanger sequencing.

**Cell culture and bacterial infection.** A549 human alveolar basal epithelial cells (ATCC CCL-185) were maintained as described (6–12), seeded to 1.5 x 10^5^ cells/well on 24-well plates for 32 h, then serum starved for 16 h before infection. NCI-H292 mucoepidermoid pulmonary human carcinoma epithelial cells (ATCC CRL-1848) were maintained as described (13), and seeded to 4 x 10^5^ cells/well on 24-well plates for 24 h before infection. For infections, PBS-normalized bacterial suspensions (OD_600_=1, ~10^9^ c.f.u./mL) were prepared using NTHi freshly grown on PVX agar for 16 h. A ~90% confluence was reached by the time of infection. To monitor bacterial invasion, a multiplicity of infection (MOI) of ~100:1 (200 µL bacterial suspension) was added in 1 mL EBSS per well (Earle's Balanced Salt Solution, Gibco), followed by 2 h incubation to allow for invasion. Wells were then washed 3 times with PBS, incubated for 1 h with RPMI 1640 (containing 10% FCS, Hepes 10 mM) and gentamicin (Gm) 200 μg/mL, and washed again 3 times with PBS. Host cells were lysed with 300 μL PBS-saponin 0.025% for 10 min at RT, and serial dilutions were plated on sHTM agar, generating 3 hpi counts. To quantify intracellular bacteria over time, infections were performed as described above; after Gm removal and washing, cells were incubated for 1 (4 hpi), 3 (6 hpi), 5 (8 hpi) or 21 h (24 hpi) with RPMI 1640 and Gm 16 μg/mL (12), before cell lysis and serial dilution plating. Results are expressed as log c.f.u./well.

**Live imaging.** For live imaging experiments, A549 cells were seeded to 2.5 x 10^4^ cells/well in 8-well tissue chambers (Sarstedt) for 32 h, and serum starved for 16 h before infection. A ~70% confluence was reached by the time of infection. Forty-five min before infection, the medium was replaced with 0.5 mL RPMI 1640 (with 10% FCS, Hepes 10 mM) per well, and A549 cells were loaded with 0.5 μM LysoTracker Red DN99 (Invitrogen). A GFP-expressing NTHi strain (86-028NP with pRSM2211) (14) was used for life imaging. PBS-normalized bacterial suspensions (OD_600_=0.2) were prepared as above. Fifteen min prior to imaging, a 1 µL suspension/well (MOI ~4:1) was added, and the 8-well chamber was mounted to the Leica DMi8 microscope onto an automated stage fully enclosed by a OKO-Lab Cage Incubator chamber preincubated at 37ºC. Live imaging with a 63x objective continued up to 1 h post-infection. Multi-positional acquisition module from Leica Application Suite X (LAS X, version 3.4.2.18368) was used to select and record 12 different fields that contained infected cells with at least one bacterium per cell. Fluorescent and DIC (differential interference contrast) images were acquired every 3 min for each selected position. GFP and LysoTracker fluorescence signals were detected using Leica filter sets 11525314 and 11525310, respectively. Images and time-lapse movies were processed and analysed with Icy (http://icy.bioimageanalysis.org) and Adobe Photoshop software packages.

**Immunofluorescence microscopy on fixed samples.** A549 cells were seeded to 7 x 10^4^ cells/well on 13 mm circular coverslips in 24-well plates for 32 h, and serum starved for 16 h prior infection. Normalized bacterial suspensions were prepared as above, and 5 µL bacterial suspension/well (MOI ~1:8) were used for infection during 2 h in 1 mL EBSS. Wells were washed 3 times with PBS and incubated in RPMI 1640 (with 10% FCS, Hepes 10 mM) and Gm (see intracellular bacterial Gm procedure). Cells were washed 3 times with PBS and fixed with 3.7% paraformaldehyde (PFA) in PBS pH 7.4 for 15 min at RT. *H. influenzae* cells were stained with a rabbit anti-NTHi serum (12) diluted 1:600. Acidic compartments were loaded with 0.5 μM LysoTracker Red DN99 45 min before PFA-fixation. Late endosomes were stained with mouse monoclonal anti-human Lamp-1 H4A3 antibody (Developmental Studies Hybridoma Bank) diluted 1:100. DNA was stained with Hoechst 33342 (Invitrogen) diluted 1:2500. Donkey anti-rabbit or donkey anti-mouse conjugated to Cy2, and donkey anti-mouse conjugated to Rhodamine secondary antibodies (Jackson Immunological) were diluted 1:100. Fluorescent *in situ* hybridization (FISH) was performed on PFA-fixed infected cells by using Cy3-labeled oligonucleotides EUB338 and GAM42a, designed for specific labeling of the rRNA of eubacteria and the gamma subclass of the proteobacteria, respectively (**Table S2**) as described (12). After staining, coverslips were washed 3 times in PBS and once in distilled water before mounting onto glass slides using ProLong Gold antifade mounting gel. Fixed cells were imaged with a Leica DMi8 fluorescence microscope and a Hamamatsu ORCA Flash 4.0LT camera. Acquired images were processed with the Icy (<http://icy.bioimageanalysis.org/>) and Adobe Photoshop software packages.

**Bacterial RNA extraction and RT-qPCR analysis.** NTHi strains were grown for 16 h on PVX chocolate agar. Two to five colonies were then inoculated into 20 mL sBHI, grown for 12 h at 100 r.p.m., diluted into 20 mL fresh sBHI to OD_600_=0.05, and grown to OD_600_=0.6 at 200 r.p.m over 2 to 4 hrs. From each culture, 7 mL were recovered, pelleted (4000 r.p.m, 4 min), flash frozen and stored at -80ºC. Total RNA was isolated using TRIzol reagent (Invitrogen) and quality was evaluated using RNA 6000 Nano LabChips (Agilent 2100 Bioanalyser). All samples had intact 16S and 23S ribosomal RNA. Reverse transcription was performed using 1 µg RNA by PrimerScript RT Reagent kit (Takara). To measure *hmw1A* and *hmw2A* gene expression*,* cDNA diluted 1:10 were used as template in a 20 µL reaction mixture containing 1X SYBR Premix Ex Taq II (Tli RNaseH Plus) (Takara), and specific primers pairs for each strain and gene (**Table S2**). Fluorescence data were analysed with AriaMx Real-Time PCR System (Agilent Technologies). Relative mRNA quantities were calculated using the comparative threshold cycle (Ct) method and normalized using *gyrA* gene expression as an endogenous control.

**SDS-PAGE and Western blotting.** To monitor HMW protein levels, whole cell extracts were prepared from bacteria recovered from PVX chocolate agar plates grown overnight into PBS and adjusted to OD_600_=1. To monitor GFP protein levels, whole cell extracts were prepared from bacteria recovered from overnight grown sBHI Erm_11_-agar plates with PBS and adjusted to OD_600_=3. In all cases, samples were diluted 1:1 with protein loading buffer, sonicated for 1 min, incubated for 5 min at 95ºC, and for 1 min on ice before gel loading. Samples were separated in SDS-PAGE 8% (HMW) or 12% (GFP) gels on a mini-PROTEAN Tetra Cell system (BioRad), and proteins stained with Coomassie Brilliant Blue R-250 staining solution (Bio-Rad), or transferred to a nitrocellulose membrane for immunoblotting. HMWA was detected by using a primary guinea pig anti-HMWA (gp85) antibody diluted 1:2000 and incubated for 90 min at RT (15), and a secondary goat anti-guinea pig IgG (Santa Cruz, sc-2438) antibody conjugated to horseradish peroxidase, diluted 1:5000 and incubated for 1 h at RT. GFP was detected by using a primary mouse anti-GFP (Living colors, Clontech) antibody diluted 1:5000 and incubated for 1 h at RT, and a secondary goat anti-mouse IgG (Thermofisher Scientific, 32430) antibody conjugated to horseradish peroxidase, diluted 1:2500 and incubated for 1 h at RT. ECL AdvanceTM Western Blotting Detection Kit (GE HealthCare) was used for development.

**Biofilm formation assays.** Three biofilm assays were used. First, biofilm biomass was detected by crystal violet staining. Overnight grown *H. influenzae* cultures were diluted to OD_600_=0.01 in fresh sBHI, transferring 150 μL/well of diluted cultures to polystyrene 96-well flat bottom plates (Sarstedt), and plate incubation at 37ºC with 5% CO_2_ for 24 h without shaking. Bulk bacterial biomass was assessed by measuring OD_600_ on a SpectraMAX 340 microplate reader. The liquid portion in each well was then discarded, plates were washed 3 times through gentle submersion in distilled water and allowed to air dry. Next, 150 μL/well 0.5% crystal violet (Sigma-Aldrich) were added, and plates were incubated for 20 min at RT, followed by plate washing as previously described. Finally, 150 μL/well 95% ethanol (Merck) were added, plates were incubated for 20 min at RT, and OD_570_ was determined as a measure of biofilm biomass. The OD_570_/OD_600_ ratio for each strain and independent assay was also calculated to normalize biofilm biomass to overall growth. Second, NTHi biofilms imaged by confocal microscopy. Cultures were grown on an eight-well chambered cover glass for confocal image analysis (Ibidi GmbH) from a starting OD_600_ of 0.01 in 2 mL of sBHI. Plates were incubated at 37ºC for 24h without shaking. After incubation, wells were washed with distilled water and stained for 15 min in the dark with the fluorescent Live/Dead BacLight Bacterial Viability Kit (Invitrogen), following the manufacturer’s instructions. Samples were washed three times to remove nonspecific staining, and fluorescence was observed by confocal laser microscopy. Z-stack images were acquired with a Carl Zeiss LSM 880 laser scanning spectral confocal microscope (Carl Zeiss Microscopy GmbH, Jena, Germany) with a distance of 0.5 microns between planes using 488 nm and 561 nm laser lines with a 40x 1.3NA immersion oil objective. Two z-stack images were randomly acquired from the central area of each well from two separate experiments, which included duplicates for each strain. The obtained images were processed with different software packages: IMARIS software (Bitplane AG, Switzerland) was used to obtain the images of 3D rendering of the different biofilms. Average thickness was calculated using the ZEN 2.3 software (Carl Zeiss Microscopy GmbH) as the average value of the biofilm height rising from the solid plate in the z direction between cross-sections. Maximum intensity projections and XZ-plane lateral projections were obtained using Fiji ImageJ (imagej.net/Fiji) software. Third, NTHi biofilms were imaged by atomic force microscopy (AFM). Cultures were grown on Thermanox™ circular coverslips placed in 24-well microtiter plates covered with sBHI medium, and incubated at 37ºC for 24 h. Afterwards, slides were washed three times in milliQ water and left to air dry prior to imaging. Samples were imaged in air using the XE-70 AFM (Park Systems, Korea) operated in non-contact mode using the ACTA (Applied NanoStructures, CA, USA) pyramidal-shaped silicon cantilevers with a nominal spring constant *k* = ±40 N/m and a nominal resonance frequency *f* = ±300 kHz. Images were acquired at a scan rate of 0.3–0.6 Hz and a scan size of 5x5 µm. At least 30 images per experiment were acquired to have a representative sample. Data were transformed into topography, signal error and phase images, and analyzed by means of the XEP and XEI software (Park Systems, Korea).

**References**

1. Mell JC, Viadas C, Moleres J, Sinha S, Fernandez-Calvet A, Porsch EA, St Geme 3rd JW, Nislow C, Redfield RJ, Garmendia J. 2016. Transformed recombinant enrichment profiling rapidly identifies HMW1 as an intracellular invasion locus in *Haemophilus influenzae*. PLoS Pathog2016/04/29. 12:e1005576.

2. Allen S, Zaleski A, Johnston JW, Gibson BW, Apicella MA. 2005. Novel sialic acid transporter of *Haemophilus influenzae*. Infect Immun 73:5291–5300.

3. Herriott RM, Meyer EM, Vogt M. 1970. Defined nongrowth media for stage II development of competence in *Haemophilus influenzae*. J Bacteriol 101:517–524.

4. Charpentier E Barry P, Alfonso B, Fang Y, Novick RP. AAI. 2004. Novel cassette-based shuttle vector system for Gram-positive bacteria. Appl Env Microbiol 70:6076–6085.

5. Catalan-Moreno A, Cela M, Menendez-Gil P, Irurzun N, Caballero CJ, Caldelari I, Toledo-Arana A. 2021. RNA thermoswitches modulate *Staphylococcus aureus* adaptation to ambient temperatures. Nucleic Acids Res https://doi.org/10.1093/nar/gkab117.

6. Euba B, Lopez-Lopez N, Rodriguez-Arce I, Fernandez-Calvet A, Barberan M, Caturla N, Marti S, Diez-Martinez R, Garmendia J. 2017. Resveratrol therapeutics combines both antimicrobial and immunomodulatory properties against respiratory infection by nontypeable *Haemophilus influenzae*. Sci Rep 7:12860.

7. Euba B, Moleres J, Segura V, Viadas C, Morey P, Moranta D, Leiva J, De-Torres JP, Bengoechea JA, Garmendia J. 2015. Genome expression profiling-based identification and administration efficacy of host-directed antimicrobial drugs against respiratory infection by nontypeable *Haemophilus influenzae*. Antimicrob Agents Chemother 59:7581–7592.

8. Euba B, Moleres J, Viadas C, Barberan M, Caballero L, Grillo MJ, Bengoechea JA, de-Torres JP, Linares J, Leiva J, Garmendia J. 2015. Relationship between azithromycin susceptibility and administration efficacy for nontypeable *Haemophilus influenzae* respiratory infection. Antimicrob Agents Chemother 59:2700–2712.

9. Fernandez-Calvet A, Rodriguez-Arce I, Almagro G, Moleres J, Euba B, Caballero L, Marti S, Ramos-Vivas J, Bartholomew TL, Morales X, Ortiz-de-Solorzano C, Yuste JE, Bengoechea JA, Conde-Alvarez R, Garmendia J. 2018. Modulation of *Haemophilus influenzae* interaction with hydrophobic molecules by the VacJ/MlaA lipoprotein impacts strongly on its interplay with the airways. Sci Rep2018/05/04. 8:6872.

10. Fernández-Calvet A, Euba B, Caballero L, Díez-Martínez R, Menéndez M, Ortiz de Solórzano C, Leiva J, Micol V, Barrajón-Catalán E, Garmendia J. 2019. Preclinical evaluation of the antimicrobial-immunomodulatory dual action of xenohormetic molecules against *Haemophilus influenzae* respiratory infection. Biomolecules 9:891.

11. Lopez-Gomez A, Cano V, Moranta D, Morey P, Garcia del Portillo F, Bengoechea JA, Garmendia J. 2012. Host cell kinases, α5 and β1 integrins, and Rac1 signalling on the microtubule cytoskeleton are important for non-typeable *Haemophilus influenzae* invasion of respiratory epithelial cells. Microbiology 158:2384–2398.

12. Morey P, Cano V, Marti-Lliteras P, Lopez-Gomez A, Regueiro V, Saus C, Bengoechea JA, Garmendia J. 2011. Evidence for a non-replicative intracellular stage of nontypeable *Haemophilus influenzae* in epithelial cells. Microbiology 157:234–250.

13. Rodriguez-Arce I, Marti S, Euba B, Fernandez-Calvet A, Moleres J, Lopez-Lopez N, Barberan M, Ramos-Vivas J, Tubau F, Losa C, Ardanuy C, Leiva J, Yuste JE, Garmendia J. 2017. Inactivation of the thymidylate synthase *thyA* in non-typeable *Haemophilus influenzae* modulates antibiotic resistance and has a strong impact on its interplay with the host airways. Front Cell Infect Microbiol 7:266.

14. Mason KM, Munson Jr. RS, Bakaletz LO. 2003. Nontypeable *Haemophilus influenzae* gene expression induced *in vivo* in a chinchilla model of otitis media. Infect Immun 71:3454–3462.

15. Buscher AZ, Grass S, Heuser J, Roth R, St Geme 3rd JW. 2006. Surface anchoring of a bacterial adhesin secreted by the two-partner secretion pathway. Mol Microbiol2006/06/15. 61:470–483.

16. Elango D, Schulz BL. 2019. Phase-variable glycosylation in nontypeable *Haemophilus influenzae*. J Proteome Res2019/11/28. https://doi.org/10.1021/acs.jproteome.9b00657.

17. Grass S, Lichti CF, Townsend RR, Gross J, St Geme 3rd JW. 2010. The *Haemophilus influenzae* HMW1C protein is a glycosyltransferase that transfers hexose residues to asparagine sites in the HMW1 adhesin. PLoS Pathog2010/06/05. 6:e1000919.

18. Gross J, Grass S, Davis AE, Gilmore-Erdmann P, Townsend RR, St Geme 3rd JW. 2008. The *Haemophilus influenzae* HMW1 adhesin is a glycoprotein with an unusual N-linked carbohydrate modification. J Biol Chem2008/07/16. 283:26010–26015.
