# Supplementary material for: Phase Variation in HMW1A Controls a Phenotypic Switch in Haemophilus influenzae Associated with Pathoadaptation during Persistent Infection
Source: mBio. 2021 Jun 22;12(3):e00789-21. doi: 10.1128/mBio.00789-21 (PMC8262952; doi:10.1128/mBio.00789-21)
Supplement: TABLE S1 [file mbio.00789-21-st001.docx]

**Table S1.** Plasmids used in this study.

| **Name** | **Description** | **Source** |
| --- | --- | --- |
| pGEM-T easy | Cloning vector (Amp^R^) | Promega |
| pGEM-T 10 c.2 | pGEM-T with a 3,093 bp insert containing part of the *hmw1A*_86-028NP_ gene and its upstream flanking region (Amp^R^) | This study |
| pBSLerm | Plasmid containing an Erm resistance cassette (Erm^R^) | (2) |
| pGEM-T  /Δ*hmw1A*::*ermC* | pGEM-T with a 4,114 bp insert containing a *hmw1A*_86-028NP_ disruption cassette (Amp^R^, Erm^R^) | This study |
| pJET1.2/blunt | Cloning vector (Amp^R^) | Thermofisher Scientific |
| pJET/*hmw1BC*-HI1680 | pJET1.2 with a 4,336 bp insert containing the *hmw1C*_86-028NP_ gene and its flaking regions (Amp^R^) | This study |
| pJET/Δ*hmw1C*::*ermC* | pJET1.2 with a 3,296 bp insert containing a  *hmw1C*_86-028NP_ disruption cassette (Amp^R^, Erm^R^) | This study |
| pMK-Pr-*hmw1A*-22rep | pMK with a 1,200 bp insert containing the *hmw1A*_86-028NP_ promoter with (SSR)_22_ (Km^R^) | This study, GeneArt Gene Synthesis, Invitrogen |
| pJET/NTHI1981-22rep-*hmw1A* | pJET1.2 with a 2,766 bp insert containing the  *hmw1A*_86-028NP_ promoter with (SSR)_22_ and its flaking regions (Amp^R^) | This study |
| pJET/NTHI1981-*ermC*-22rep-*hmw1A* | pJET1.2 with a 3,954 bp insert containing a  *hmw1A*_86-028NP_ promoter replacement cassette (Amp^R^, Erm^R^) | This study |
| pRSM2211 | Plasmid containing the *gfpmut3* gene whose expression is controlled by the promoter of the *ompP2* gene (Km^R^) | (14) |
| pCN47 | *E. coli-S. aureus* shuttle vector for cloning. (Amp^R^-Erm^R^) | (4) |
| pACYC177 | Cloning vector (Cm^R^) | New England Biolabs |
| pHRG | pCN47 plasmid containing the P*hyper* constitutive promoter, *icaR* RBS and *gfp* reporter gene | (5) |
| pTBH-01 | pACYC177 derivative containing an Erm^R^ gene and a MCS (Amp^R^, Erm^R^) | This study |
| pTBH03-*P_hmw_* | Reporter plasmid, pTBH-01 derivative containing a *Pr_hmw_::gfp* transcriptional fusion | This study |
| pJET1.2*-*P2-(SSR)_13_-P1 | pJET1.2 with a 400 bp insert containing the *P_hmw1A-_*_86-028NP_ promoter (P2-(SSR)_13_-P1), amplified from rRdS / P540 genomic DNA with primers P-hmw1A_Fw_SphI_v2 / #2082 and hmw1A_Rv_EcoRI_v2 / #2083 (Amp^R^) | This study |
| pJET1.2-P2-(SSR)_24_-P1 | pJET1.2 with a 470 bp insert containing the *r_hmw1A_*_-86-028NP_ promoter (P2-(SSR)_24_-P1), amplified from rRdS-24 / P1066 genomic DNA with primers P-hmw1A_Fw_SphI_v2 / #2082 and hmw1A_Rv_EcoRI_v2 / #2083 (Amp^R^) | This study |
| pJET1.2*-*P2-(SSR)_14_ | pJET1.2 with a 332 bp insert containing a *P_hmw1A-_*_86-028NP_ promoter region (P2-(SSR)_14_), amplified from rRdS / P540 genomic DNA with primers P-hmw1A_Fw_SphI_v2 / #2082 and Rep_Rv_EcoRI / #2084 (Amp^R^) | This study |
| pJET1.2-P2-(SSR)_24_ | pJET1.2 with a 402 bp insert containing a *P_hmw1A-_*_86-028NP_ promoter region (P2-(SSR)_24_), amplified from rRdS-24 / P1066 genomic DNA with primers P-hmw1A_Fw_SphI_v2 / #2082 and Rep_Rv_EcoRI / #2084 (Amp^R^) | This study |
| pJET1.2-(SSR)_14_-P1 | pJET1.2 with a 222 bp insert containing a *P_hmw1A-_*_86-028NP_ promoter region ((SSR)_14_-P1), amplified from rRdS / P540 genomic DNA with primers P-Rep_Fw_SphI / #2085 and hmw1A_Rv_EcoRI_v2 / #2083 (Amp^R^) | This study |
| pJET1.2-(SSR)_24_-P1 | pJET1.2 with a 292 bp insert containing a *P_hmw1A-_*_86-028NP_ promoter region ((SSR)_24_-P1), amplified from rRdS-24 / P1066 genomic DNA with primers P- Rep_Fw_SphI / #2085 and hmw1A_Rv_EcoRI_v2 / #2083 (Amp^R^) | This study |
| pTBH03-P2-(SSR)_13_-P1 | pJET1.2*-*P2-(SSR)_13_-P1 was *Sph*I/*EcoR*I digested and the excised insert ligated into pTBH03-*Pr_hmw_* digested with the same restriction enzymes (Amp^R^, Erm^R^) | This study |
| pTBH03-P2-(SSR)_24_-P1 | pJET1.2*-*P2-(SSR)_24_-P1 was *Sph*I/*EcoR*I digested and the excised insert ligated into pTBH03-*Pr_hmw_* digested with the same restriction enzymes (Amp^R^, Erm^R^) | This study |
| pTBH03-P2-(SSR)_14_ | pJET1.2*-*P2-(SSR)_14_ was *Sph*I/*EcoR*I digested and the excised insert ligated into pTBH03-*Pr_hmw_* digested with the same restriction enzymes (Amp^R^, Erm^R^) | This study |
| pTBH03-P2-(SSR)_24_ | pJET1.2-P2-(SSR)_24_ was *Sph*I/*EcoR*I digested and the excised insert ligated into pTBH03-*Pr_hmw_* digested with the same restriction enzymes (Amp^R^, Erm^R^) | This study |
| pTBH03-(SSR)_14_-P1 | pJET1.2-(SSR)_14_-P1 was *Sph*I/*EcoR*I digested and the excised insert ligated into pTBH03-*Pr_hmw_* digested with the same restriction enzymes (Amp^R^, Erm^R^) | This study |
| pTBH03-(SSR)_24_-P1 | pJET1.2-(SSR)_24_-P1 was *Sph*I/*EcoR*I digested and the excised insert ligated into pTBH03-*Pr_hmw_* digested with the same restriction enzymes (Amp^R^, Erm^R^) | This study |
| pTBH03-P2 | pTBH03-P2-(SSR)_13_-P1 was used as a template for inverse PCR with primers P2_Hmw1A_EcoRI / #2087 and P1_Hmw1A_SphI / #2086. The PCR fragment was digested with *EcoR*I and religated (Amp^R^, Erm^R^) | This study |
| pTBH03-P1 | pTBH03-P2-(SSR)_13_-P1 was used as a template for inverse PCR with primers P2_Hmw1A_EcoRI / #2087 and P1_Hmw1A_SphI / #2086. The PCR fragment was digested with *Sph*I and religated (Amp^R^, Erm^R^) | This study |
